# Characterization of the single-subunit oligosaccharyltransferase STT3A from *Trypanosoma brucei* using synthetic peptides and lipid-linked oligosaccharide analogs

**DOI:** 10.1093/glycob/cwx017

**Published:** 2017-03-16

**Authors:** Ana S Ramírez, Jérémy Boilevin, Rasomoy Biswas, Bee Ha Gan, Daniel Janser, Markus Aebi, Tamis Darbre, Jean-Louis Reymond, Kaspar P Locher

**Affiliations:** 2 Institute of Molecular Biology and Biophysics, Eidgenössische Technische Hochschule (ETH), CH-8093 Zürich, Switzerland; 3 Department of Chemistry and Biochemistry, University of Berne, CH-3012 Berne, Switzerland; 4 Institute of Microbiology, Eidgenössische Technische Hochschule (ETH), CH-8093 Zürich, Switzerland

**Keywords:** enzyme kinetics, lipid-linked oligosaccharide, *N*-glycosylation, oligosaccharyltransferase

## Abstract

The initial transfer of a complex glycan in protein *N*-glycosylation is catalyzed by oligosaccharyltransferase (OST), which is generally a multisubunit membrane protein complex in the endoplasmic reticulum but a single-subunit enzyme (ssOST) in some protists. To investigate the reaction mechanism of ssOST, we recombinantly expressed, purified and characterized the STT3A protein from *Trypanosoma brucei* (TbSTT3A). We analyzed the in vitro activity of TbSTT3A by synthesizing fluorescently labeled acceptor peptides as well as lipid-linked oligosaccharide (LLO) analogs containing a chitobiose moiety coupled to oligoprenyl carriers of distinct lengths (C_10_, C_15_, C_20_ and C_25_) and with different double bond stereochemistry. We found that in addition to proline, charged residues at the +1 position of the sequon inhibited glycan transfer. An acidic residue at the −2 position significantly increased catalytic turnover but was not essential, in contrast to the bacterial OST. While all synthetic LLO analogs were processed by TbSTT3A, the length of the polyprenyl tail, but not the stereochemistry of the double bonds, determined their apparent affinity. We also synthesized phosphonate analogs of the LLOs, which were found to be competitive inhibitors of the reaction, although with lower apparent affinity to TbSTT3A than the active pyrophosphate analogs.

## Introduction

Protein *N*-glycosylation confers a multitude of functions to the acceptor macromolecules, facilitating diverse interactions and signaling pathways (Kelleher and Gilmore 2006; Breitling and Aebi 2013; Xu and Ng 2015; Cherepanova et al. 2016). The initial transfer of the glycan moiety from a lipid-linked oligosaccharide (LLO) donor to the asparagine of the acceptor protein is catalyzed by oligosaccharyltransferase (OST), an enzyme located in the membrane of the endoplasmic reticulum  in eukaryotic cells or in the plasma membrane of bacteria.

The mechanism of OST-catalyzed *N*-glycosylation has been investigated using the bacterial PglB protein, a single subunit OST (ssOST) from *Campylobacter lari*. Structural and in vitro functional studies of PglB have provided insight into peptide recognition and have identified catalytically important residues (Schwarz et al. 2010; Lizak et al. 2011, 2013, 2014; Gerber et al. 2013). In contrast to bacterial, eukaryotic OSTs have different levels of complexity, varying between different species (Cherepanova et al. 2016). In fungi and vertebrates, OST is a membrane complex composed of up to eight subunits, whereas in insects and plants it is predicted to be composed of seven subunits. The OST enzymes of certain protists are predicted to have six subunits (i.e., *Cryptosporidium parvum*), four subunits (i.e., *Plasmodium falciparum*) or even a single subunit as in kinetoplastids (i.e., *Trypanosoma brucei* and *Leishmania major*) (Kelleher and Gilmore 2006; Nasab et al. 2008). Intriguingly, certain kinetoplastids contain several orthologues of ssOSTs that originate from gene duplication. The existence of more than one ssOST has been proposed to allow a wider range of acceptor sequons to be glycosylated (Izquierdo et al. 2009; Schwarz and Aebi 2011). *T. brucei* contains three distinct orthologues of ssOST, denoted TbSTT3A, TbSTT3B and TbSTT3C. Two of them, TbSTT3A and TbSTT3B, are endogenously expressed at different stages of the life cycle of the parasite, whereas expression of TbSTT3C was not detected (Izquierdo et al. 2009). In vivo studies suggested that TbSTT3A and TbSTT3B have different preferences for the LLO donor as well as for acceptor sequon (Jones et al. 2005; Manthri et al. 2008). Whereas TbSTT3B requires a c-branch in the LLO (present in Man_9_GlcNAc_2_-PP-Dol) and glycosylates sequons surrounded by neutral or basic residues, TbSTT3A preferentially transfer glycans from LLO donors lacking the c-branch such as Man_5_GlcNAc_2_-PP-Dol to sequons surrounded by acidic side chains (Izquierdo et al. 2009, 2012).

The structural and mechanistic basis of substrate recognition and preference in ssOST enzymes is unknown. Structural and functional studies are needed to rationalize the in vivo function of eukaryotic OST enzymes. Mechanistic studies using purified eukaryotic OST enzymes are scarce, which is in part due to the challenges associated with their expression and purification. Therefore, much of the structural and mechanistic insight has been derived from the bacterial (PglB) and archaeal (AglB) ssOST enzymes (Lizak et al. 2011, 2014; Gerber et al. 2013; Matsumoto et al. 2013; Liu et al. 2014). Protist ssOST enzymes offer an opportunity to study eukaryotic OST with the reduced complexity of a single membrane protein. Here we describe the heterologous expression, purification and functional analysis of the *T. brucei* ssOST enzyme TbSTT3A. We generated fluorescently labeled acceptor peptides and varied the residues surrounding the glycosylation sequon to increase the affinity and turnover of the reaction. We also synthesized various LLO analogs with polyprenyl chains of controlled length and coupled to chitobiose. In addition, we generated inhibitory LLO analogs where the pyrophosphate group was replaced with an unreactive pyrophosphonate moiety. This allowed us to perform detailed in vitro studies on TbSTT3A, providing functional insight into the mechanism of eukaryotic ssOST.

## Results

### Expression and purification of TbSTT3A

**Fig. 1.**
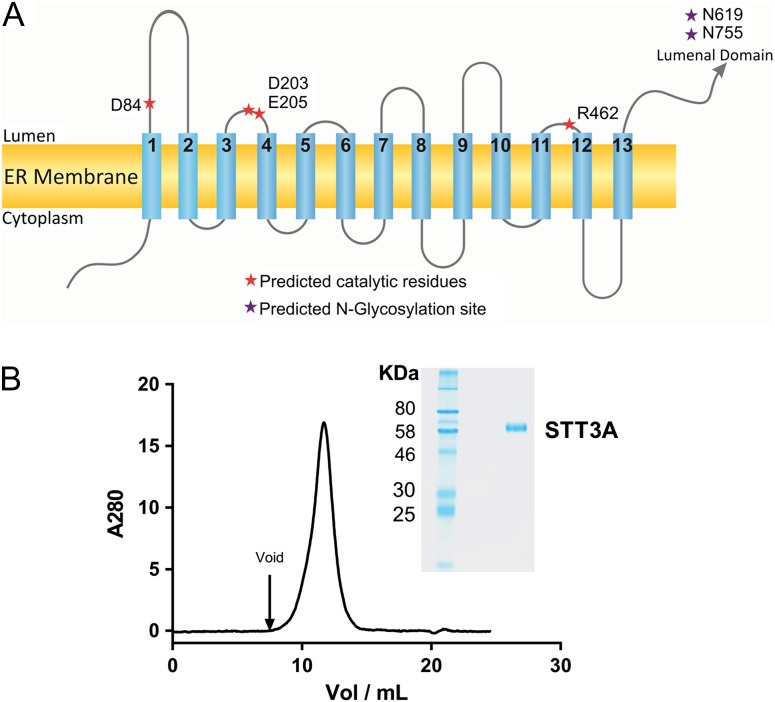
(**A**) Schematic representation of the predicted transmembrane topology of TbSTT3A. The positions of the expected catalytic residues based on sequence alignment with PglB are shown as red asterisks. Purple asterisks depict the two glycosylation sites in the luminal domain of TbSTT3A (N619 and N765). (**B**) SEC and SDS-PAGE analysis of purified TbSTT3A. 85 µg of purified and deglycosylated TbSTT3A were loaded on a Superdex S200 column at 0.5 mL/min. SDS-PAGE analysis of purified TbSTT3A is shown in the inset. 1.7 µg of purified TbSTT3A was loaded on a 10% polyacrylamide gel. A single band is observed for the purified protein. This figure is available in black and white in print and in color at *Glycobiology* online.

To identify the most suitable STT3 orthologue for functional and structural studies, we screened 13 genes encoding ssOST enzymes from protists (Parsaie Nasab et al. 2013): *Leishmania braziliensis* (*LbSTT3A*, *LbSTT3B*, *LbSTT3C*), *Leishmania infantum* (*LiSTT3A*, *LiSTT3B*, *LiSTT3C*), *L. major* (*LmSTT3A*, *LmSTT3B*, *LmSTT3C*, *LmSTT3D*) and *T. brucei* (*TbSTT3A*, *TbSTT3B*, *TbSTT3C*). Expression constructs were generated by fusing either a His_10_-YFP tag to the N-terminus or a YFP-His_10_ tag to the C-termini of the proteins. All constructs were heterologously expressed in HEK293 cells. Expression levels and protein monodispersity after extraction with detergent were evaluated by fluorescent size exclusion chromatography. TbSTT3A showed the highest expression level and was selected for all subsequent biochemical analyses. Even though TbSTT3A shares 81% identity with TbSTT3B and TbSTT3C, the expression of these last two was significantly lower. The main difference between TbSTT3A and its orthologs is the length of its C-terminal domain, which is 23 amino acids shorter in TbSTT3A compared to TbSTT3B and TbSTT3C, this might influence the recombinant expression of the proteins. According to topology predictions, TbSTT3A has the same transmembrane topology as the bacterial OST, PglB (Figure 1A). Although TbSTT3A and PglB share a low percentage of sequence similarity (15.4% identity, 28.1% similarity), some of the catalytic residues of PglB are conserved (Figure 1A and Supplementary Fig. 1).

Large-scale overexpression of TbSTT3A with His_10_-YFP fused to its N-terminus was performed in Sf9 cells. Various parameters, in particular the choice and concentration of the detergent used to solubilize and purify the protein, were carefully screened and optimized. Purified and deglycosylated TbSTT3A ran as a single band in SDS-PAGE electrophoresis and showed a single peak in size exclusion chromatography, with a small shoulder at higher mass, indicating potential aggregation (Figure 1B). The obtained yield and purity allowed detailed functional investigation of TbSTT3A and will enable us to pursue structural studies in the future.

### Synthesis and in vitro glycosylation of acceptor peptides

**Fig. 2.**
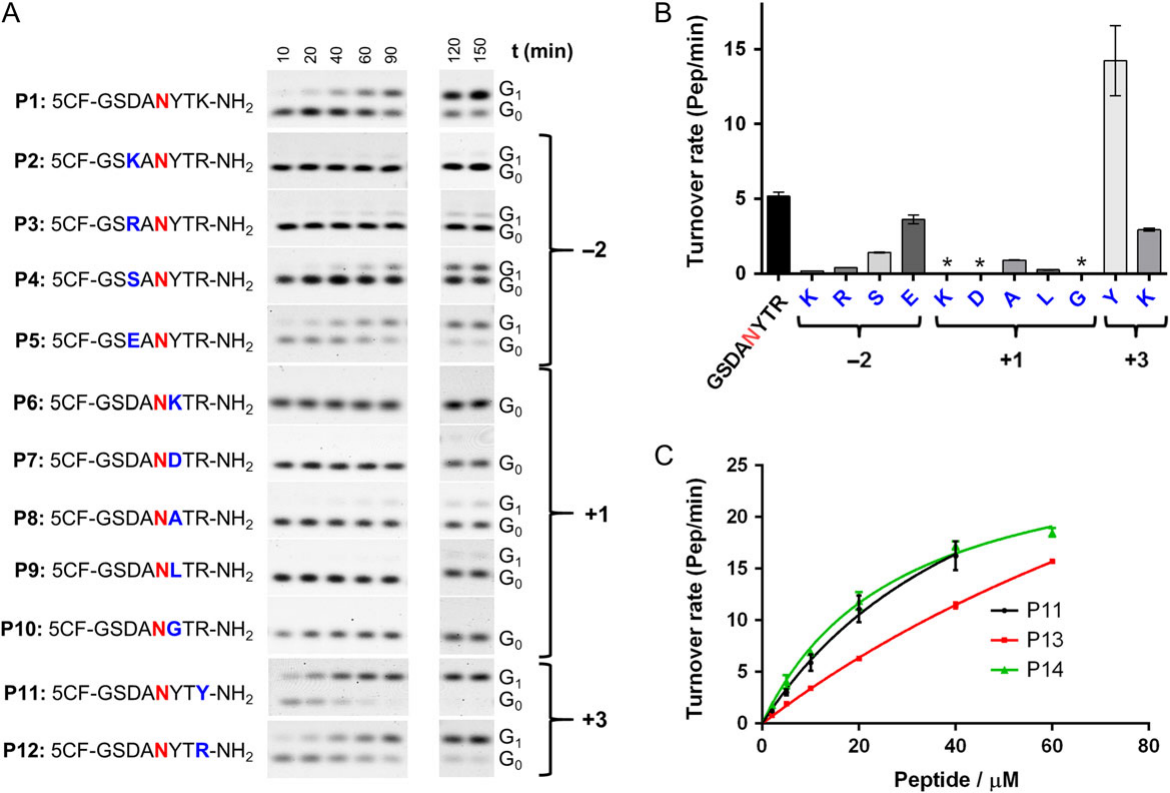
Optimization of the acceptor peptide sequence. (**A**) Synthetic acceptor peptides are shown in single letter code. The acceptor asparagine (zero position of the sequon) is indicated in red. Glycosylation experiments were performed and the fluorescently labeled substrate (G_0_) and product (G_1_) were quantified following Tricine SDS-PAGE analysis. (**B**) Turnover rates after fitting the time points by linear regression using PRISM. Error bars indicate standard error of the fitting. (**C**) Kinetic analysis of substrate peptides: glycosylation experiments were performed with 20 nM purified TbSTT3A, 50 µM farnesyl-PP-chitobiose, 10 mM MnCl_2_, 150 mM NaCl, 20 mM Hepes pH 7.5, 0.035% DDM, 0.007% CHS and different concentrations of peptides varying from 2 to 60 µM. Data points reflect the mean of three separate measurements. Error bars indicate standard deviations. Data were fitted by nonlinear regression according to the Michaelis–Menten formula using PRISM. This figure is available in black and white in print and in color at *Glycobiology* online.

Following previously reported assay designs used for in vitro studies of bacterial PglB and archaeal AglB (Kohda et al. 2007; Gerber et al. 2013; Liu et al. 2014), we synthesized a range of peptides containing a glycosylation sequon and a 5-carboxyfluorescein label that was attached to the N-terminus for quantitation purposes. Starting from the peptide sequence DANYTK, which was previously used to study PglB and the cytoplasmic glycosyltransferase NGT (Schwarz et al. 2011; Liu et al. 2014), we explored how distinct side chains in and around the sequon influenced the activity of TbSTT3A (Figure 2A and B). In vitro glycosylation experiments were performed with purified TbSTT3A and farnesyl-PP-chitobiose **1b** (see below) as a donor substrate (Figure 2A and B).

Analysis of different residues at the −2 position revealed that highest turnover rates were obtained with negatively charged side chains (5.2 ± 0.3 for aspartate and 3.6 ± 0.3 pep/min for glutamate respectively). In contrast, the positively charged side chains arginine or lysine caused a decrease in the turnover rate of ~28- and ~12-fold, respectively (Figure 2B). The small, polar residue serine at the −2 position caused a 3.7-fold decrease in turnover. Analysis of different residues at the +1 position of the sequon showed that besides proline, other amino acids strongly inhibited glycosylation by TbSTT3A. Charged residues such as lysine or aspartate were not tolerated. Nonpolar residues such as alanine and leucine led to a pronounced decrease in turnover (5.8- and 20-fold, respectively, compared to tyrosine) and glycine led to no detectable glycosylation under the conditions tested. The highest turnover was observed for a tyrosine residue at this position (Figure 2B). At the +3 position of the sequon, an additional tyrosine residue resulted in the highest turnover compared to any other amino acids inserted at this position. Lysine or arginine were also tolerated at the +3 position, but the turnover rate was 4.9- and 2.7-fold lower when compared to that of tyrosine-containing sequon.

Among the peptides screened in this study, P11 showed the highest turnover rate (Figure 2B). Unfortunately, kinetic analysis of P11 did not allow accurate determination of *K*_*M*_ and *k*_cat_ values because saturation was not reached, even at the highest peptide concentrations experimentally possible (Figure 2C). To test whether an increase in peptide length might affect the affinity and in vitro glycosylation efficiency of P11, we synthesized peptides with two additional residues, either at the N-terminus (P13) or at the C-terminus (P14) (Table I). Elongation of the C-terminus led to an increase in peptide affinity, and a *K*_*M*_ value of 28 ± 3 µM could be determined (Figure 2C). The opposite was observed when the peptide was elongated at the N-terminus: In this case, it was not possible to determine *K*_*M*_ and *k*_*ca*t_ values, because there was an almost linear response of the observed activity with increasing peptide concentration (Figure 2C, Table I). The combined changes in the sequence, from the initial DANYTK to GSDANYTYTQ, led to an increase of about 3-fold in glycosylation turnover and a significant increase of the apparent affinity (*K*_*M*_: 28 ± 3 µM). Therefore, peptide P14 was used in all subsequent assays.

**Table I.** Kinetic parameters for the synthetic substrate peptides

Peptide	*K*_*M*_ (µM)	*k_cat_*(pep/min)
P11: 5CF-GSDANYTY	50 ± 10^a^	37 ± 5^a^
P13: 5CF-GSGSDANYTY	ND	ND
P14: 5CF-GSDANYTYTQ	28 ± 3	28 ± 1

^a^For peptide P11, accurate determination of *K*_*M*_ and *k*_cat_ values is not possible because higher peptide concentrations could not be reached due to solubility limitations.

ND: not determined. For this peptide, the apparent *K*_*M*_ value estimated is higher than the measured concentrations of peptide.

### Synthesis and kinetic analysis of LLO analogs

**Fig. 3.**
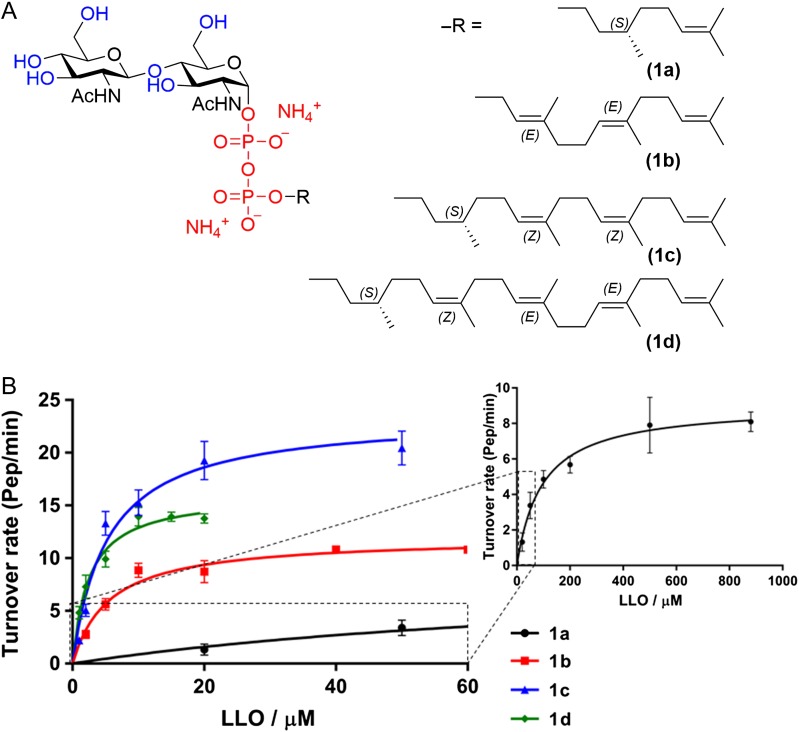
(**A**) Structure of the LLO analogs synthesized (**1a**) (*S*)-Citronellyl-PP-Chitobiose, C10; (**1b**) farnesyl-PP-chitobiose, C15; (**1c**) (*S*)-NerylCitronellyl-PP-chitobiose, C20; (**1d**) (*S*)-farnesylCitronellyl-PP-chitobiose, C25. (**B**) Kinetic analysis of synthetic LLO analogs. Glycosylation experiments were performed with 20 nM purified TbSTT3A, 10 µM peptide P14, 10 mM MnCl2, 150 mM NaCl, 20 mM Hepes pH 7.5, 0.035% DDM, 0.007% CHS and different concentrations of synthetic LLO analogs. Data points reflect the mean of 3 separate measurements. Error bars indicate standard deviations. Data were fitted by nonlinear regression according to the Michaelis–Menten formula using PRISM software. This figure is available in black and white in print and in color at *Glycobiology* online.

The native LLO in trypanosome is Dol-PP-GlcNAc_2_-Man_9_, but in vivo studies have shown that TbSTT3A preferentially uses Dol-PP-GlcNAc_2_-Man_5_ as a substrate (Izquierdo et al. 2009). Native LLOs are difficult to extract in sufficient quantities and their solubilities are low because of the long polyprenyl chain of dolichol. In contrast to the bacterial PglB enzyme, for which milligram amounts of pure LLO can be isolated by introducing the *C. jejuni pgl* operon into *Escherichia coli* SCM6 cells (Kowarik et al. 2006), such an approach is not available for the production of eukaryotic LLO. We therefore synthesized LLO analogs as donor substrates, starting from procedures reported earlier (Fang et al. 1995; Kočovský et al. 1999; Liu et al. 2014) but improving reaction conditions (see below). We synthesized four distinct analogs of the eukaryotic LLO and systematically varied the length of the lipid moiety (C_10_, C_15_, C_20_ and C_25_) as well as the double bond stereochemistry (Figure 3A). The lipid carrier in compound **1b (***E,E*-farnesol), has a double bond in the first isoprenyl unit, whereas the lipid carriers in compounds **1a**, **1c** and **1d** are structurally similar to the native dolichol. These four polyprenyl chains were coupled to a chitobiose moiety, which has been reported to be recognized and transferred by the OST complex from yeast microsomes (Fang et al. 1995).

**Fig. 4.**
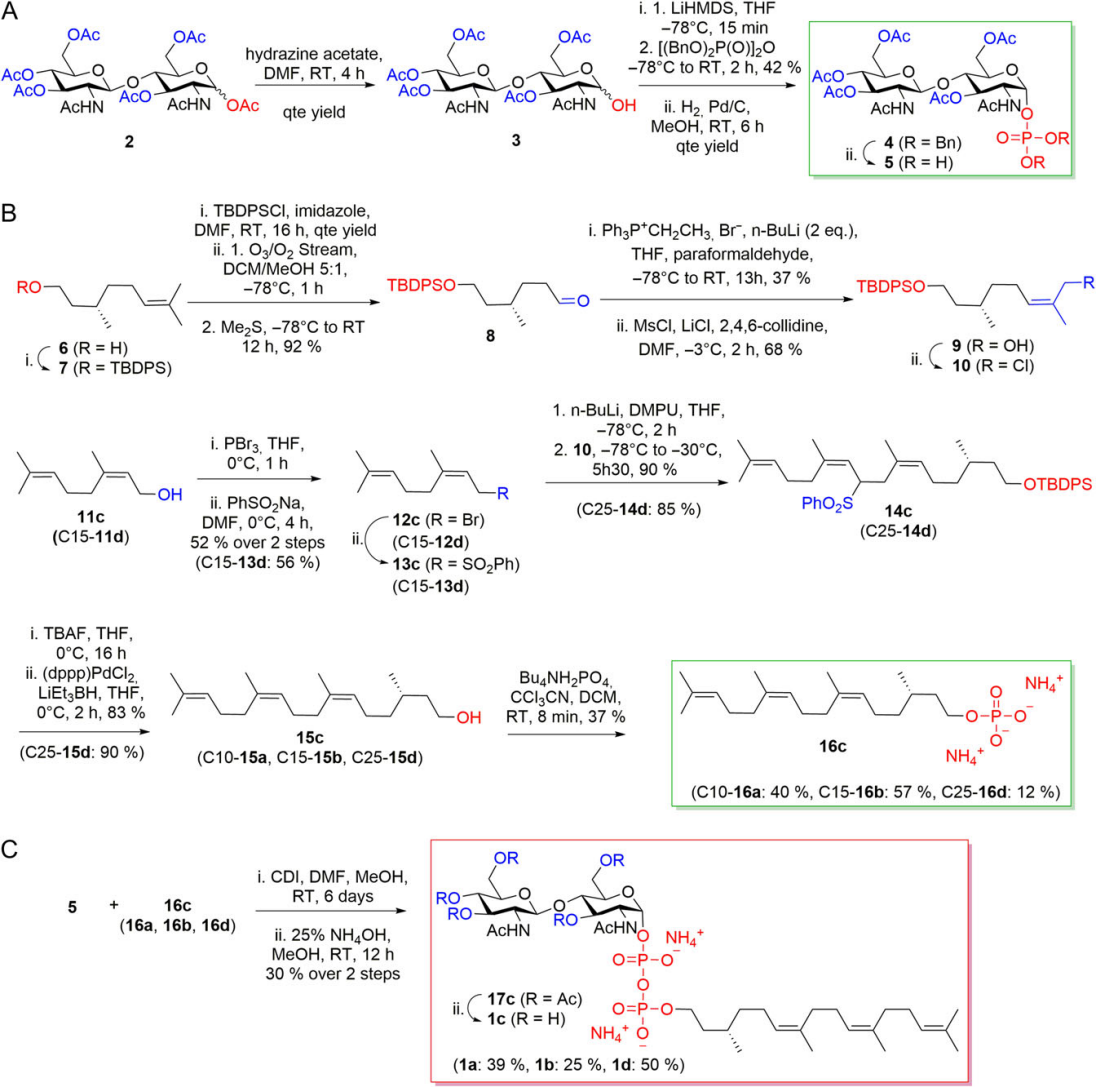
Synthesis of the LLO analogs used in this study. (**A**) Synthesis of Chitobiose phosphate precursor (**5**). (**B**) Synthesis of lipid phosphate precursors (**16a–d**). (**C**) Coupling reaction and deacetylation of monophosphates **16a–d** and **5** to final pyrophosphates **1a–d**. Synthetic pathway is shown for **1c**, yields and precursor names for the synthesis of **1a**, **1b** and **1d** are shown in parentheses. This figure is available in black and white in print and in color at *Glycobiology* online.

The synthesis of LLO analogs included three distinct steps: The synthesis of the chitobiose phosphate and of the lipid phosphate precursors, were performed in parallel and were followed by a coupling step and deacetylation of monophosphates to the final pyrophosphate (Figure 4). The peracetylated *N*,*N*′-diacetylchitobiose phosphate **5** was obtained from the commercially available *N*,*N*′-diacetylchitobiose hexaacetate **2** as described earlier (Lee and Coward 1992) (Figure 4A). Lipids **15c** and **15d** were synthesized in nine steps from (*S*)-citronellol **6**. The key intermediate **10** was prepared in 23% overall yield from silyl protected (*S*)-citronellol **7** by reductive ozonolysis to aldehyde **8**, stereoselective olefination (Kočovský et al. 1999) to form alcohol **9**, and chlorination. Intermediate **10** was then coupled with either the phenylsulfonyl nerol **13c** or the phenylsulfonyl *E,E*-farnesol **13d**, followed by desilylation and reductive removal to afford the desired lipids **15c**, **d**. Phosphorylation of the four lipids **15a–d** gave precursors **16a–d** (Figure 4B). The lipid phosphates **16a–d** were then activated with 1,1′-carbonyldiimidazole and coupled to **5** to yield after purification by chromatography and deacetylation pure LLOs **1a–d** (Figure 4C).

All of our synthetic LLO analogs were recognized as substrates by TbSTT3A. As illustrated in Figure 3B, the length of the polyprenyl chain had a significant effect on the apparent affinity (*K*_*M*_ value, Table II). The highest *K*_*M*_ value was observed for the LLO analog containing only two isoprenyl units **(1a**, *K*_*M*_ of 95 ± 18 µM), whereas LLO analogs with 3, 4 or 5 isoprenyl units (**1b**, **1c**, **1d**) resulted in lower *K*_*M*_ values (5.6 ± 0.8 µM, 5.3 ± 0.9 µM and 2.5 ± 0.3 µM, respectively). The glycosylation rate also increased with the length of the polyprenyl chain, reaching a maximum when the LLO analog contained four isoprenyl units (C_20_). Although the length of the lipid tail had an impact on the LLO affinities by TbSTT3A, the difference between the *k_cat_* values calculated for each LLO analog was maximum 2-fold (**1a**, *k_cat_*: 9.1 ± 0.5 pep/min and **1c**, *k_cat_*: 23 ± 1 pep/min), indicating that the efficiency of glycosylation was not drastically affected by the length of the lipid tail (Table II).

**Table II.** Kinetic parameters for the different synthetic LLO analogs and inhibitors

LLO analog	*K*_*M*_ (µM)	*k_cat_*(pep/min)
(1a) Citronellyl-PP-chitobiose	95 ± 18	9.1 ± 0.5
(1b) Farnesyl-PP-chitobiose	5.6 ± 0.8	12 ± 1
(1c) NerylCitronellyl-PP-chitobiose	5.3 ± 0.9	23 ± 1
(1d) FarnesylCitronellyl-PP-chitobiose	2.5 ± 0.3	16 ± 1
Inhibitory LLO	IC_50_ (µM)	
(34b) Farnesyl-PP-C-chitobiose	167 ± 14	
(34d) FarnesylCitronellyl-PP-C-chitobiose	26 ± 3	

We also tested whether a synthetic LLO analog with a single glycan moiety, farnesyl-PP-GlcNAc, could be a substrate for TbSTT3A. Previous studies showed that farnesyl-PP-GlcNAc stimulated ATPase activity of the bacterial flippase PglK in detergent (Perez et al. 2015), and the compound is also accepted as an in vitro substrate of PglB (data not shown). In our studies, no glycopeptide product was observed when farnesyl-PP-GlcNAc was used, even after overnight incubation at vast excess substrate concentrations (Supplementary Fig. 2). This indicates that two GlcNAc units is the smallest glycan moiety required by TbSTT3A to perform *N*-glycosylation.

### Synthesis and kinetic analysis of inhibitory LLO analogs

**Fig. 5.**
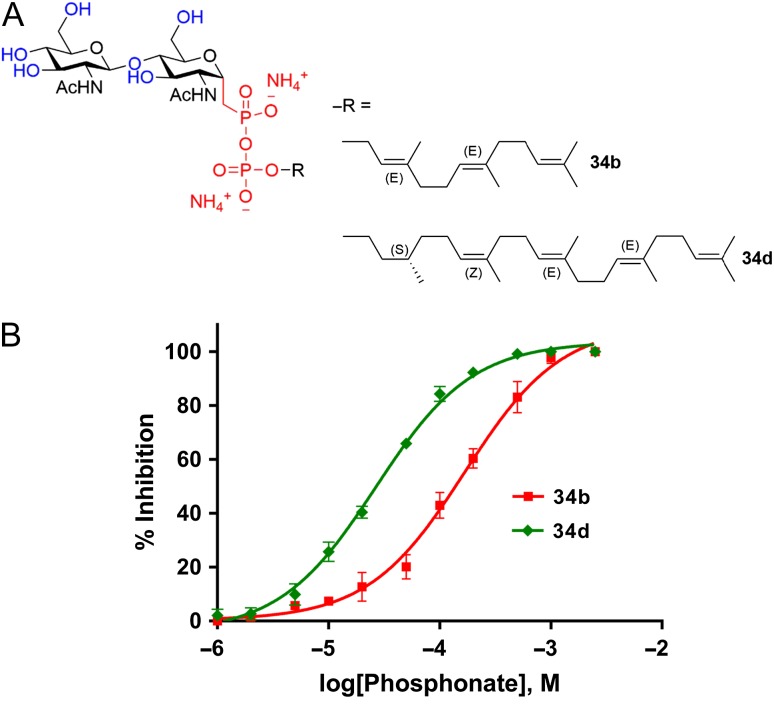
(**A**) Structure of the LLO inhibitors synthesized **(34b)** farnesyl-PP-C-chitobiose, C_15_; (**34d**) (*S*)-farnesylCitronellyl-PP-C-chitobiose, C_25_. (**B**) Kinetic analysis of synthetic LLO inhibitors. Glycosylation experiments were performed with 20 nM TbSTT3A protein, 10 µM peptide P11, 10 mM MnCl_2_, 150 mM NaCl, 20 mM Hepes pH 7.5, 0.035% DDM and 0.007% CHS. Samples were incubated with LLO competitive inhibitors (1–2500 µM) for 5 min before adding 15 µM of farnesyl-PP-chitobiose, **1b**. Data points reflect the mean of three separate measurements. Error bars indicate standard deviations. Data were fitted by nonlinear regression using PRISM software. This figure is available in black and white in print and in color at *Glycobiology* online.

Inhibitors based on nonhydrolyzable substrates analogs have been extensively studied for glycosyltransferases that use nucleotide activated sugars (Compain and Martin 2001). Among those, substrate analogs bearing a phosphonate group at the anomeric carbon of the reducing-end or first sugar moiety (Gordon et al. 2006) or an elongated sugar–phosphate bond (Luengo and Gleason 1992; Schäfer and Thiem 2000) have been described in the literature. In the case of transglycolases, which use lipid-linked sugars as substrate donors, similar inhibitors have been synthesized coupling phosphonate or elongated phosphate sugars to short lipid carriers (Qiao and Vederas 1993; Garneau et al. 2004; Lin et al. 2015). In analogy to these approaches, we designed and synthesized potentially inhibitory LLO analogs containing a pyrophosphonate moiety, but otherwise structurally identical to the substrate LLO analogs described above. We chose the lipid (*S*)*,Z,E,E*-farnesylcitronellol from the LLO analog **1d**, which showed the highest apparent affinity (lowers Km value) in TbSTT3A assays , as well as the shorter lipid *E,E*-farnesol from **1b**, which has a higher solubility in water and showed an apparent affinity only 2-fold lower compared to **1d** (Table II). The resulting compounds **34b** and **34d** (Figure 5A) contain an unreactive pyrophosphonate group instead of the pyrophosphate moiety.

**Fig. 6.**
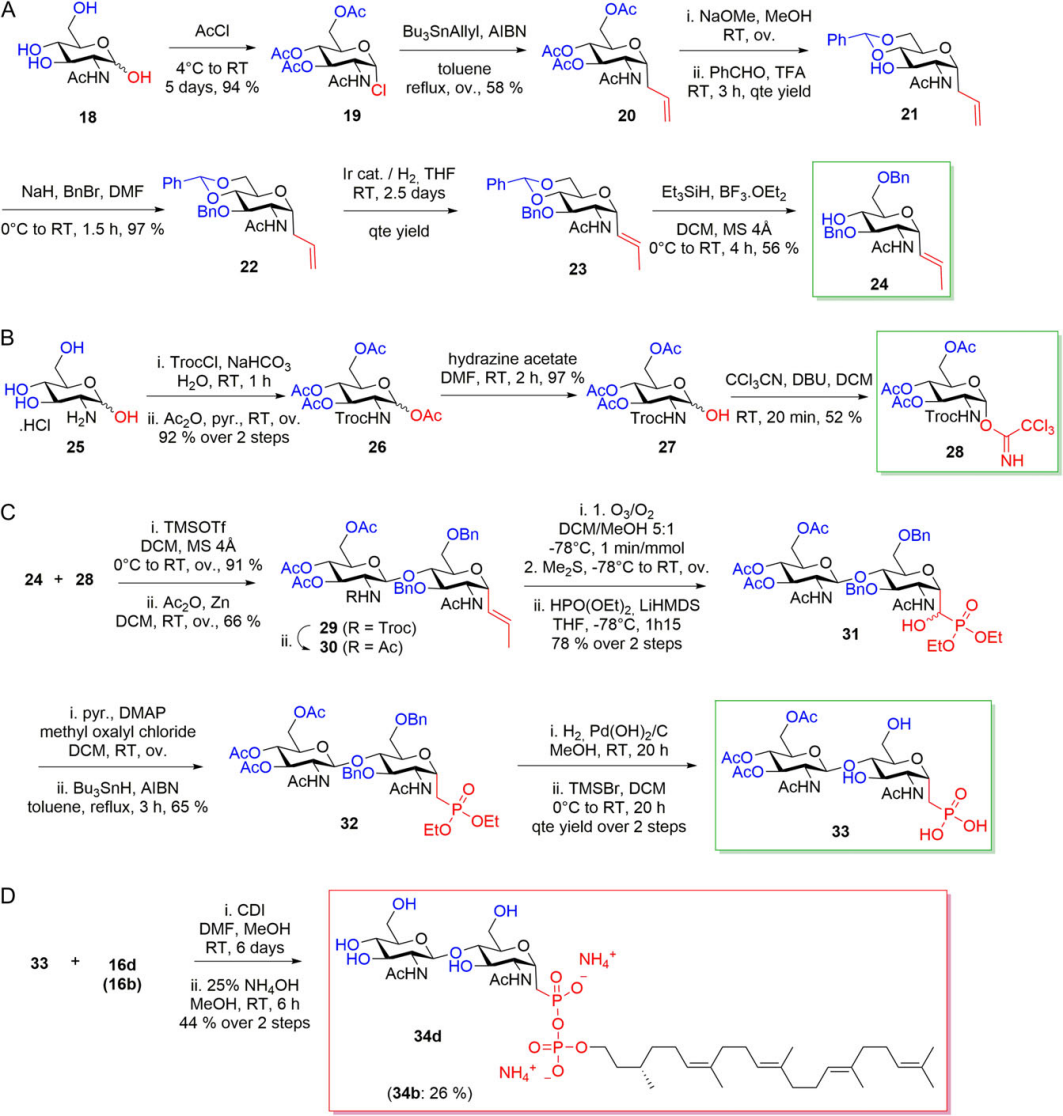
Synthesis of LLO inhibitors **34b** and **34d. **(**A**) Preparation of glycosyl acceptor precursor **24. **(**B**) Synthesis of glycosyl donor precursor **28. **(**C**) Preparation of chitobiose phosphonate precursor **33. **(**D**) Coupling reaction and deacetylation of monophosphates **16b** and **16d** with **33** to final pyrophosphates **34b** and **34d**. Synthetic pathway is shown for **34d**, yields and precursor names for the synthesis of **34b** are shown in parentheses. This figure is available in black and white in print and in color at *Glycobiology* online.

The synthesis of the pyrophosphonate analogs required 22 steps for compound **34b** and 31 steps for compound **34d** from commercially available starting materials. The glycosyl acceptor **24** was obtained in seven steps from *N*-acetyl-D-glucosamine by acetylation/chlorination, radical allylation, deacetylation, orthogonal protection of position 4 and 6 with benzaldehyde, benzyl protection of position 3, allyl double bond isomerization and selective deprotection of position 4 (Bouvet and Ben 2006) (Figure 6A). The glycosyl donor **28** was obtained in 4 steps from glucosamine hydrochloride as described earlier (Dullenkopf et al. 1996) (Figure 6B). The coupling reaction between glycosyl acceptor **24** and donor **28** formed the disaccharide **29**, which was converted into chitobiose phosphonate **32** and finally chitobiose phosphonic acid **33** (Lin et al. 2015) (Figure 6C). Lipid phosphates **16b** and **16d** were then activated with 1,1′-carbonyldiimidazole and coupled with **33** to yield pure LLOs, **34b** and **34d**, after deacetylation and purification by chromatography in moderate yields (Figure 6D).

Both compounds **34b** and **34d** inhibited TbSTT3A-catalyzed glycosylation of acceptor peptides under the conditions tested. Similar to the observations with reactive LLO analogs discussed above, the length of the polyprenyl chain affected the apparent affinity of the inhibitors (Figure 5B, Table II). The IC_50_ value obtained with compound **34b** was 167 ± 14 µM, whereas that of compound **34d** was 26 ± 3 µM. However, both inhibitors show a significantly lower apparent affinity (IC_50_ vs. *K*_*M*_) compared to their reactive, pyrophosphate-containing counterparts.

## Discussion

Single subunit OSTs have been mostly characterized in vivo and by complementation studies in yeast in which the endogenous *STT3* gene or other essential components of the multimeric OST complex were deleted. These studies have shown that some ssOSTs could replace the catalytic subunit STT3 (Castro et al. 2006; Izquierdo et al. 2009) or even the whole octameric OST complex from yeast (Nasab et al. 2008; Hese et al. 2009). Intriguingly, ssOSTs from *T. brucei* have been shown to exhibit different preferences towards LLO donor and sequon acceptors in vivo despite sharing high sequence identity (Izquierdo et al. 2009, 2012). In vitro studies kinetically characterizing ssOSTs have been scarce because of the challenges associated with their purification in functional form from the membrane as well as the low availability of substrates. Our work describes the first large-scale recombinant expression, purification and characterization of a eukaryotic ssOST.

Optimization of a substrate peptide for our in vitro studies was achieved by analyzing the effect of variations in the amino acids at position −2, +1 and +3 of the sequon on glycosylation turnover and the *K_M_* values. We found that the highest rates were obtained when an acid residue was present at position −2. The crystal structure of PglB with bound substrate peptide revealed that the strict requirement of an acidic residue at the −2 position of the sequon was due to the formation of a stabilizing salt bridge with a positively charged R331 conserve in bacterial PglB homologs (Lizak et al. 2011). A residue equivalent to R331 from PglB has not been found in eukaryotic orthologues of STT3 (including TbSTT3A) which may explain the more relaxed specificity of the protein towards amino acids at the −2 position. We found that the activity of TbSTT3A is strongly inhibited by presence of charged residues or, surprisingly, a glycine at the +1 position of the sequon. This contrasts with findings described for PglB and for the eukaryotic octameric OST complex, where the only amino acid not tolerated at the +1 position was proline. Our finding suggests that TbSTT3A has additional structural requirements for the acceptor peptide, at least in vitro.

Generating sufficiently large amounts of functional LLO analogs was one of the challenges for the development of in vitro studies with eukaryotic OSTs. Our approach of chemically synthesizing functional LLO analogs was of paramount importance for studying the interactions of ssOST with the LLO substrate. Our results showed that several synthetic LLO analogs can serve as glycan donor substrates of TbSTT3A in vitro, which is in line with findings reported for the yeast OST complex (Fang et al. 1995). The length of the isoprenyl tail seems to be an important determinant of the apparent affinity, but does not influence the glycosylation efficiency (turnover) of TbSTT3A (Table II), suggesting that it is not involved in catalytic turnover, as it was previously reported for PglB (Liu et al. 2014) and the *O*-oligosaccharyltransferase PglL (Musumeci et al. 2013). Intriguingly, we did not observe a significant effect of the presence or absence of a double bond in the first isoprenyl unit of LLO on turnover, nor of the stereochemistry of the polyprenyl tail in the affinity of TbSTT3A for the LLO analogs, suggesting that there is little specificity in the interaction of TbSTT3A and the polyprenyl tail of bound LLO.

In contrast to farnesyl-PP-chitobiose, which is recognized as a substrate by TbSTT3A with a remarkably high apparent affinity (*K*_*M*_: 5.6 ± 0.8 µM), farnesyl-PP-GlcNac, which contained only a single sugar moiety, was not recognized as a substrate. This suggests that two GlcNAc units are the minimal glycan structure that can be processed by TbSTT3A in vitro, which contrasts with earlier studies performed with partially purified octa-subunit OST complex from pig liver (Bause et al. 1995) as well as from yeast (Tai and Imperiali 2001). In both these studies, it was concluded that octameric OST could recognize Dol-PP-GlcNAc as a substrate in vitro, albeit as a poor donor.

The design and characterization of phosphonate inhibitors for TbSTT3A was performed in analogy to previous approaches reported for transglycosylases (Qiao and Vederas 1993; Garneau et al. 2004; Lin et al. 2015). We synthesized two phosphonate LLO analogs by varying the length of the polyprenyl tail. Both inhibitory LLOs carried a phosphonate group coupled to chitobiose. Both pyrophosphonate LLO analogs inhibited TbSTT3A in vitro, although the affinities of the inhibitors were lower compared to their substrate counterparts, which might indicate that the presence of the methylene group instead of the oxygen in the natural pyrophosphate bond has an influence in the binding by TbSTT3A. Intriguingly, for the bacterial transglycosylase (TGase), lipid II analogs carrying a phosphonate group did not inhibit the protein in vitro, but the presence of an elongated sugar phosphate bond instead led to inhibition (IC_50_: 25 μM) (Lin et al. 2015).

In summary, our results showed the in vitro substrate requirements of a eukaryotic ssOST enzyme. Purified TbSTT3A is active and our synthetic compounds faithfully represent native LLOs in our functional studies. Finally, these LLO analogs together with the characterized inhibitors will facilitate future structural studies aimed at visualizing LLO-bound states of OST at high resolution.

## Materials and methods

### Screening of different STT3 orthologues

The genes encoding the STT3 orthologues from *L. braziliensis* (*STT3A*, *STT3B*, *STT3C*), *L. infantum* (*STT3A*, *STT3B*, *STT3C*), *L. major* (*STT3A*, *STT3B*, *STT3C*, *STT3D*) and *T. brucei* (*STT3A*, *STT3B*, *STT3C*) were amplified from previously reported constructs (Parsaie Nasab et al. 2013) and cloned into a modified pUC57 vector carrying either a His_10_-YFP tag at the N-terminus or a YFP-His_10_ tag at the C-terminus. Expression of genes fused to YFP was screened by transient transfection in human embryonic kidney (HEK293) cells, followed by harvesting, resuspension in lysis buffer (25 mM Tris pH 8.0, 250 mM NaCl, 10% glycerol) and solubilization with a mixture of 1% w/v *N*-dodecyl-β-d-Maltopyranoside (DDM), 0.2% w/v cholesteryl hemisuccinate Tris Salt (CHS, Anatrace). Solubilized samples were analyzed by fluorescence size exclusion chromatography using a TSKgel G3000SWxl Column (TOSHO)(Kawate and Gouaux 2006).

### Expression and purification of TbSTT3A

A synthetic gene encoding TbSTT3A, optimized for expression in insect cells was purchased from Thermo Fischer Scientific. It was fused to a N-terminal His_10_-YFP tag, and cloned into pOET1 vector (Oxford Expression Technologies). Baculovirus production was performed using *flash*BAC DNA (Oxford Expression Technologies) in *Spodoptera frugiperda* (Sf9) insect cells following the manufacturer's instructions. Infected Sf9 cells were cultured in serum-free SF4 medium (Amimed) at 27 °C for 60 h. Cells were collected by centrifugation at 6500 × *g* and washed with phosphate-buffered saline. Cell pellets were frozen in liquid nitrogen and stored at −80°C until the time of use. For purification, cell pellets were thawed and resuspended in lysis buffer (25 mM K_2_HPO4/NaH_2_PO_4_, pH 7.0; 250 mM NaCl; 10% w/v Glycerol) supplemented with cOmplete™, EDTA-free Protease Inhibitor Cocktail (Roche). Lysis was performed by dounce homogenization on ice and the cell lysate was incubated with 1% (w/v) DDM, 0.2% (w/v) CHS for two hours at 4°C, then submitted to high-speed centrifugation (35,000 rpm, Ti45i rotor, 30 min). All subsequent buffers contained 0.035% (w/v) DDM, 0.007% (w/v) CHS. The supernatant was loaded onto a Ni/NTA super flow affinity column (Qiagen), washed with the same lysis buffer but containing 50 mM imidazole and eluted with the same buffer but containing 200 mM imidazole. The protein was desalted into 20 mM phosphate, pH 7.0; 150 mM NaCl; 5% glycerol (v/v) using a HiPrep 26/10 column (GE Healthcare) and incubated with home-produced 3C protease and EndoF1 endoglycosidase overnight at 4°C (Walker et al. 1994). His_10_-YFP, 3C and EndoF1 were removed by incubation with Ni/NTA super flow. TbSTT3A was further purified by size exclusion chromatography (Superdex 200 10/300 GL, GE Healthcare) in desalting buffer and peak fractions were pooled and concentrated to 2 µM for subsequent functional studies. The removal of the glycans (leaving a single GlcNAc moiety in each glycosylated residue) was confirmed by mass spectrometry (Chen et al. 2013).

### Synthesis of acceptor peptides labeled with 5-carboxyfluorescein

Peptide synthesis was carried out either manually or with the CEM Liberty Microwave automated peptide synthesizer. Manually, the synthesis was initiated by loading TentaGel S RAM resin (300 or 500 mg, loading: 0.25 or 0.26 mmol/g) in a 10-mL polypropylene syringe fitted with a polypropylene frit, a teflon stopcock and a stopper. The resin was swollen in DCM (5 mL, 20 min). After removal of the DCM, the Fmoc-protecting group of the resin was removed by using a solution of 20% piperidine in NMP. Stirring of the reaction mixture at any given step was performed by attaching the closed syringes to a rotating axis. The completion of the reaction was checked using the TNBS test. Removal of the Fmoc-protecting group of the attached amino acid was performed by using a solution of 20% piperidine in NMP (5 mL, 2 × 10 min). After filtration, the resin was washed with NMP (3 × 4 mL^2^), MeOH (3 × 4 mL^2^) and DCM (3 × 4 mL^2^). Coupling of amino acids was performed by using Fmoc-protected amino acids (3 eq), PyBOP (3 eq) and DIPEA (6 eq) in NMP (5 mL). The resin was stirred for 2 h before it was washed three times with 4 mL NMP, MeOH and DCM. Acetylation of the resin was performed after each amino-acid coupling by using a solution of acetic anhydride/DCM1:1. The synthesis performed on CEM Liberty Blue was based on Fmoc solid phase peptide chemistry. The synthesis was carried out by using Rink Amide MBHA resin (100–200 mesh), unloaded (0.78 mmol/g), 0.25 mM scale (150 mg of resin). The coupling reaction was performed in microwave at 75°C (40 W microwave power, 1 × 360 s) with the following parameters: 5 eq (relative to resin loading) of Fmoc amino acid and Oxyma in DMF and 5 eq DIC. Fmoc deprotection was performed using the default instrument protocol (20% piperidine in DMF, 1 × 180 s) at 75°C (100 W microwave power). The resin was then removed from the instrument and transferred to a synthesis syringe for manual coupling of the 5-carboxyfluorescein. Coupling of 5-CF was performed by using 5-CF (2 eq), HOBt (5 eq) and DIC (5 eq) in NMP (5 mL). The resin was stirred overnight and protected from light using aluminum foil, washed with NMP, MeOH and DCM (3 × 4 mL^2^ each). A solution of 20% piperidine in NMP (8 × 4 mL^2^, until the supernatant was colorless) was added to the resin to remove the excess of free 5-CF before the resin was finally washed with DCM (5 × 4 mL^2^). After coupling of the fluorophore, the compounds were protected from light with aluminum foil at all time. Trifluoroacetic acid (TFA) cleavage was performed by adding a solution of TFA/TIS/H_2_O (94:5:1, v/v/v) to the resin for 1–2 h. The peptide was precipitated with t-BuOMe and dissolved in H_2_O/MeCN with 0.1% TFA mixture. Labeled acceptor peptides were purified by preparative RP-HPLC. Analytical RP-UPLC was performed in Dionex ULTIMATE 3000 Rapid Separation LC System (ULTIMATE-3000RS diode array detector) using Dionex Acclaim^®^ RSLC 120 C18 column (2.2 μm, 120 Å, 3.0 × 50 mm^2^, flow 1.2 mL/min). Compounds were detected by UV absorption at 214 nm. Data recording and processing was performed with Dionex Chromeleon Management System Version 6.80 (analytical RP-HPLC). Preparative RP-HPLC was performed either with a Waters PrepLC4000 chromatography or Waters Prep 150 LC system using a Dr. Maish GmbH Reprospher Column (C18-DE, 5 μm, 100 × 30 mm^2^, pore size 100 Å, flow rate of 60 mL/min). Compounds were detected by UV absorption at 214 nm using a Waters 486 Tunable Absorbance detector. All RP-HPLC were using HPLC-grade acetonitrile and Milli-Q deionized water. The elution solutions were: **A**: H_2_O with 0.1% TFA; **B**: H_2_O/MeCN (50:50); **C**: H_2_O/MeCN (10:90) with 0.1% TFA; **D**: H_2_O/MeCN (40:60) with 0.1% TFA. MS spectra, recorded on a Thermo Scientific LTQ OrbitrapXL were provided by the Mass Spectrometry service of the Department of Chemistry and Biochemistry at the University of Berne. Characterization of each individual peptide by mass spectrometry can be found in Supporting information.

### Chemical synthesis of LLO analogs and inhibitors

A detailed description of the synthesis of the LLO analogs can be found in Supporting information**.**

### In vitro glycosylation assay

Reaction mixtures contained 20 nM purified TbSTT3A protein, 50µM farnesyl-PP-GlcNAc or farnesyl-PP-chitobiose, 10 mM MnCl_2_, 150 mM NaCl, 20 mM Hepes pH 7.5, 0.035% DDM, 0.007% CHS and 20 µM of the peptide. Reactions were incubated at 30°C. Samples were taken at different time points and reactions were stopped by the addition of 4X SDS Laemmli buffer. Samples were diluted 200-fold prior to analysis by Tricine SDS-PAGE consisting of a 16% resolving gel with 6 M urea, a 10% spacer gel and a 4% stacking gel (Schägger 2006). Fluorescent bands for peptide and glycopeptide were visualized by using a Typhoon Trio Plus imager (GE Healthcare) with excitation set at 488 nm and using a 526 nm SP emission filter. The amount of formed glycopeptide was determined from band intensities of fluorescence gel scans (ImageJ) (Gerber et al. 2013). Turnover rate determination was performed by fitting of the data to linear regression using PRISM software.

For Michaelis–Menten kinetics of the peptides, reaction mixtures containing 20 nM TbSTT3A protein, 50 µM farnesyl-PP-chitobiose, 10 mM MnCl_2_, 150 mM NaCl, 20 mM Hepes pH 7.5, 0.035% DDM and 0.007% CHS were incubated with varying concentrations (2–60 µM) of peptides P11, P13 and P14. Samples were taken at different time points and the turnover rate of the reactions were determined as before. Data were fitted by nonlinear regression according to the Michaelis–Menten formula using PRISM software.

For Michaelis–Menten kinetics of the LLO analogs, reaction mixtures containing 20 nM TbSTT3A protein, 10 µM peptide P11, 10 mM MnCl_2_, 150 mM NaCl, 20 mM Hepes pH 7.5, 0.035% DDM and 0.007% CHS were incubated with different concentrations of LLO analogs (10–900 µM, for **1a**; 2–60 µM, for **1b**; 1–50 µM, for **1c** and 1–20 µM, for **1d**). Samples were taken at different time points and turnover rates of the reactions were determined as before. Data were fitted by nonlinear regression according to the Michaelis–Menten formula using PRISM software.

For inhibition analysis, reaction mixtures containing 20 nM TbSTT3A protein, 10 µM peptide P11, 10 mM MnCl_2_, 150 mM NaCl, 20 mM Hepes pH 7.5, 0.035% DDM and 0.007% CHS were incubated with different concentrations of LLO competitive inhibitors (1–2500 µM) for 5 min at 30°C. Reactions were started with addition of 15 µM of farnesyl-PP-chitobiose, **1b**. Samples were taken at different time points and turnover rates of the reactions were determined as before. Data were fitted to nonlinear regression and IC_50_ was determined using PRISM software.

## Supplementary Material

Supplementary Data

## Abbreviations

OST, oligosaccharyltransferase; ssOST, single-subunit oligosaccharyltransferase; LLO, lipid linked oligosaccharide.
