# Supplementary material for: Characterization of the single-subunit oligosaccharyltransferase STT3A from Trypanosoma brucei using synthetic peptides and lipid-linked oligosaccharide analogs
Source: Glycobiology. 2017 Mar 16;27(6):525–35. doi: 10.1093/glycob/cwx017 (PMC5421464; doi:10.1093/glycob/cwx017)
Supplement: Supplementary Data [file Supplementaryinformation_revision.docx]

**Supporting Information for:**

***In vitro* N-glycosylation of acceptor peptides using synthetic lipid-linked oligosaccharides reveals substrate specificity of eukaryotic single-subunit oligosaccharyltransferase from *Trypanosoma brucei***

**Ana S. Ramírez^1†^, Jérémy Boilevin^2†^, Rasomoy Biswas^2§^, Bee Ha Gan^2^, Daniel Janser^1^, Markus Aebi^3^, Tamis Darbre^2^, Jean-Louis Reymond^2^ and Kaspar P. Locher^1^***

^1^ Institute of Molecular Biology and Biophysics, Eidgenössische Technische Hochschule (ETH), CH-8093 Zürich, Switzerland.

^2^ Department of Chemistry and Biochemistry, University of Berne, CH-3012 Berne, Switzerland.

^3^Institute of Microbiology, Eidgenössische Technische Hochschule (ETH), CH-8093 Zürich, Switzerland.

**Keywords:** N-Glycosylation, Oligosaccharyltransferase, Lipid-linked oligosaccharide, Enzyme Kinetics.

**†** Both authors contributed equally to this work.

* To whom correspondence should be addressed: Dept. of Biology, Institute of Molecular Biology and Biophysics, ETH Zurich, Schafmattstrasse 20, 8093 Zurich, Switzerland. Tel.: 41-44-633–3991; Fax: 41-44-633-1182; E-mail: [locher@mol.biol.ethz.ch](mailto:locher@mol.biol.ethz.ch).

§ Present Address: Aenova Holding GmbH, 82319 Starnberg, Germany.

**Supplementary Figures:**

**
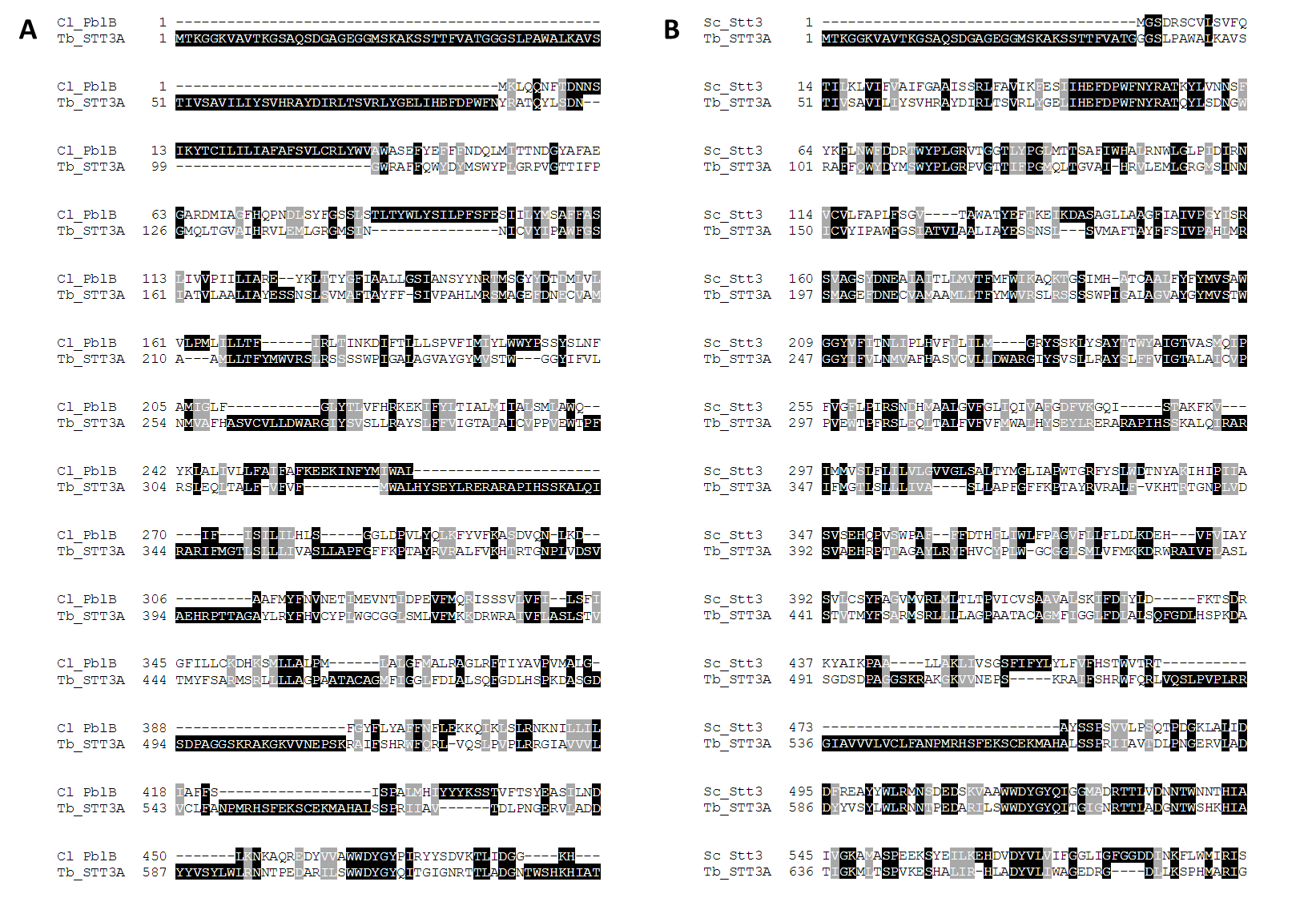
**

**Supplementary Figure 1: (A)** Sequence alignment of Stt3A from *Trypanosoma brucei* and PglB from *Campylobacter lari*. **(B)** Sequence alignment of Stt3A from *Trypanosoma brucei* and Stt3 from *Saccharomyces cerevisiae*

**
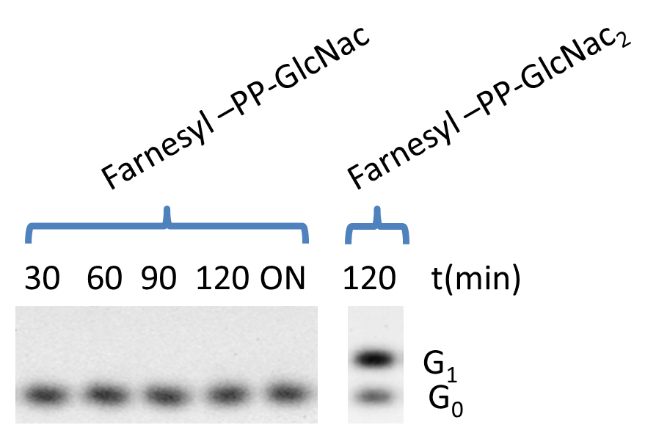
**

**Supplementary Figure 2: Glycosylation Assays with farnesyl-PP-GlcNAc.** *In vitro* glycosylation experiments were performed with 20nM purified *Tb*Stt3A purified protein, 50µM Farnesyl-PP-GlcNAc or Farnesyl-PP-GlcNac_2_, 10 mM MnCl_2_, 150 mM NaCl, 20 mM Hepes pH 7.5, 0.035% DDM, 0.007% CHS and 10 µM of the peptide P1.

**Supplementary Table 1:** HPCL-MS analysis for the synthetic peptides

| **Peptide** | **HRMS** |
| --- | --- |
| P1: 5-CF-GSDANYTR | (ESI+) calc. for C_56_H_65_N_13_O_20_ [M+H]^+^: 1241.2; obsd: 1240. 5, [M+2H]^2+^: 621.1, obsd: 621.2. |
| P2: 5CF-GSKANYTR | (ESI+) calc. for C_58_H_72_N_14_O_18_ [M+H]^+^: 1254.3; obsd: 1254.5, [M+2H]^2+^: 627.7, obsd: 627.7. |
| P3: 5CF-GSRANYTR | (ESI+) calc. for C_58_H_72_N_16_O_18_ [M+H]^+^: 1282.3; obsd: 1281.5, [M+2H]^2+^: 641.7, obsd: 641.8. |
| P4: 5CF-GSSANYTR | (ESI+) calc. for C_55_H_65_N_13_O_19_ [M+H]^+^: 1213.2; obsd: 1212.5, [M+2H]^2+^: 607.1, obsd: 607.2. |
| P5: 5CF-GSEANYTR | (ESI+) calc. for C_57_H_67_N_13_O_20_ [M+H]^+^: 1255.2; obsd: 1254.5,[M+2H]^2+^: 628.1, obsd: 627.54. |
| P6: 5CF-GSDANKTR | (ESI+) calc. for C_53_H_68_N_14_O_19_, [M+2H]^2+^: 603.6, obsd: 603.25; [M+3H]^3+^: 402.7, obsd: 402.5 |
| P7: 5CF-GSDANDTR | (ESI+) calc. for C_51_H_61_N_13_O_21_ [M+H]^+^: 1193.1; obsd: 1193.4, [M+2H]^2+^: 597.0, obsd: 597.2. |
| P8: 5CF-GSDANATR | (ESI+) calc. for C_50_H_61_N_13_O_19_ [M+H]^+^: 1149.1; obsd: 1148.4, [M+2H]^2+^: 575.0, obsd: 575.2. |
| P9: 5CF-GSDANLTR | HRMS (ESI+) calc. for C_53_H_67_N_13_O_19_ [M+H]^+^: 1190.2; [M+2H]^2+^: 596.1, obsd: 596.2. |
| P10: 5CF-GSDANGTR | (ESI+) calc. for C_49_H_59_N_13_O_19_ [M+H]^+^: 1135.1; obsd: 1135.4, [M+2H]^2+^: 568.0, obsd: 568.2. |
| P11: 5CF-GSDANYTY | (ESI+) calc. for C_59_H_62_N_10_O_21_ [M+H]^+^: 1246.2; obsd: 1245.0,[M+2H]^2+^: 622.6, obsd: 622.2. |
| P12: 5-CF-GSDANYTK | (ESI+) calc. for C_56_H_65_N_11_O_20_ [M+H]^+^: 1213.2; obsd: 1212.45, [M+2H]^2+^: 607.1, obsd: 606.7. |
| P13: GSGSDANYTY | (ESI+) calc. for C_64_H_69_N_11_O_25_ [M+H]^+^: 1392.31; obsd: 1391.47, [M+2H]^2+^: 697.15, obsd: 696.24; [M+2H+Na]^2+^: 706.16, obsd: 707.23; ; [M+2H+K]^2+^: 714.15, [M+3H+K]^3+^: 475.7, obsd: 477.14. |
| P14: 5CF-GSDANYTYTQ | (ESI+) calc. for C_68_H_76_N_12_O_26_ [M+H]^+^: 1477.41.44; obsd: 1476.53, [M+H+Na]^+^: 1499.41.44; obsd: 1498.51, [M+2H]^2+^: 739.71, obsd: 738.77; [M+2H+Na]^2+^:748.71, obsd: 749.75; [M+2H+K]^2+^: 756.7, obsd: 757.74; [M+3H+K]^3+^: 504.14, obsd: 505.5 |

**Supplementary Methods**

***Synthesis and characterization of LLO analogs***

**(2R,3S,4R,5R,6S)-5-acetamido-6-(((2R,3S,4R,5R,6S)-5-acetamido-4-acetoxy-2-(acetoxymethyl)-6-hydroxytetrahydro-2H-pyran-3-yl)oxy)-2-(acetoxymethyl)tetrahydro-2H-pyran-3,4-diyl diacetate (3)**

**2** (596 mg, 0.88 mmol, 1.00 eq) and hydrazine acetate (97.35 mg, 1.06 mmol, 1.20 eq) were dissolved in 5 ml DMF and stirred under Ar at RT for 4 h. The reaction mixture was then concentrated *in vacuo* to yield **3** (558 mg, 0.88 mmol, quantitative yield) as a colorless powder (DCM/MeOH 95:5, Rf = 0.06). ^1^H NMR (300 MHz, CDCl_3_) δ = 7.34-7.38 (m, 1H, H-OH), 7.21 (d, *J* = 10.2 Hz, 1H, H-1a), 6.27-6.29 (m, 1H, H-N2b), 5.61-5.67 (m, 1H, H-N2a), 5.20 (d, *J* = 3.3 Hz, 1H, H-3a), 4.96-5.11 (m, 3H, H-3b, H-4b, H-1b), 4.42 (dd, *J* = 12.3 Hz, 3.9 Hz, 1H, H-2a), 4.26-4.32 (m, 2H, H-6a1, H-6b1), 4.16-4.19 (m, 2H, H-6a2, H-6b2), 4.00-4.05 (m, 2H, H-2b, H-5b), 3.67 (d, *J* = 9.6 Hz, 1H, H-4a), 3.56-3.59 (m, 1H, H-5a), 2.12, 2.08, 2.05, 2.01, 2.00, 2.00, 1.93, 1.91 (7×s, 7×3H, 7×OAc). ^13^C NMR (75 MHz, CDCl_3_) δ = 172.0, 171.3, 171.3, 171.1, 170.9, 170.5, 169.2 (7×s, 7×C=OCH_3_), 102.2 (s, C-1b), 91.7 (s, C-1a), 72.1 (s, C-4a), 71.9 (s, C-5a), 71.1 (s, C-3a), 71.1 (s, C-3b), 68.2 (s, C-5b), 67.9 (s, C-4b), 62.5 (s, C-6a), 61.7 (s, C-6b), 54.2 (s, C-2a), 51.9 (s, C-2b), 23.1, 22.9 (2×s, 2×NHC=OCH_3_), 21.0, 20.7, 20.7, 20.6, 20.5 (5×s, 5×OC=OCH_3_). ESI-MS (+) *m/z* 635.23 (M+[H^+^]), calculated 635.23 for C_26_H_39_N_2_O_16_.

**(2R,3S,4R,5R,6S)-5-acetamido-6-(((2R,3S,4R,5R,6R)-5-acetamido-4-acetoxy-2-(acetoxymethyl)-6-((bis(benzyloxy)phosphoryl)oxy)tetrahydro-2H-pyran-3-yl)oxy)-2-(acetoxymethyl)tetrahydro-2H-pyran-3,4-diyl diacetate (4)**

**3** (558 mg, 0.88 mmol, 1.00 eq) was dissolved in 30 ml anhydrous THF, cooled down to -78°C and LiHMDS (0.97 ml, 0.97 mmol, 1.10 eq, 1M in THF) was added dropwise under Ar. After 5 min, a solution of tetrabenzyl pyrophosphate (592.3 mg, 1.10 mmol, 1.25 eq) in 3 ml THF was added dropwise. The reaction mixture was subsequently warmed up to 0°C and stirred 2 h at this temperature. Then, the solution was concentrated under reduced pressure. A flash column chromatography on silica gel basified with a few drops of a 25 % ammonium hydroxide solution in water (DCM/MeOH 98:2) was made to yield **4** (271 mg, 0.30 mmol, 42 % calculated after isolation of 0.15 mmol (96 mg) of the substrate) as a colorless powder (DCM/MeOH 95:5, Rf = 0.32). ^1^H NMR (300 MHz, CDCl_3_) δ = 7.35-7.37 (m, 10H, H-Bn), 5.91 (d, *J* = 8.7 Hz, 1H, H-1a), 5.59-5.63 (m, 2H, H-N2b, H-N2a), 5.00-5.24 (m, 7H, 2×CH_2_-Bn, H-3a, H-3b, H-4b), 4.55 (d, *J* = 8.4 Hz, H-1b), 4.42 (dd, *J* = 12.3 Hz, 4.2 Hz, 1H, H-2a), 4.25-4.31 (m, 2H, H-6a1, H-6b1), 4.03-4.07 (dd, *J* = 12.3 Hz, 1.5 Hz, 1H, H-2b), 3.92-4.02 (m, 2H, H-6a2, H-6b2), 3.78-3.87 (q, 1H, H-5b), 3.71 (t, *J* = 9.3 Hz, 1H, H-4a), 3.58-3.63 (m, 1H, H-5a), 2.07 (s, 2×3H, 2×NHC=OCH_3_), 2.02, 2.01, 2.00, 2.00, 1.93, 1.69 (5×s, 5×3H, 5×OC=OCH_3_). ^13^C NMR (75 MHz, CDCl_3_) δ = 171.0, 170.9, 170.7, 170.5, 170.3, 170.3, 169.3 (7×s, 7×C=OCH_3_), 135.3 (s, C-1a-Bn, C-1b-Bn), 128.8 (s, C-4a-Bn, C-5a-Bn, C-4b-Bn, C-5b-Bn), 128.1 (s, C-6a-Bn, C-6b-Bn), 128.0 (s, C-2a-Bn, C-3a-Bn, C-2b-Bn, C-3b-Bn), 101.2 (s, C-1b), 96.1 (s, C-1a), 75.61 (s, C-4a), 72.4 (s, C-5a), 71.9 (s, C-3a), 70.7 (s, C-3b), 69.9 (s, 2×CH_2_-Bn), 68.1 (s, C-5b), 61.8 (s, C-4b), 61.4 (s, C-6a), 54.9 (s, C-6b), 52.0 (s,C-2a), 51.9 (s, C-2b), 23.1, 22.7 (2×s, 2×NHC=OCH_3_), 20.9, 20.7, 20.6, 20.6, 20.6 (5×s, 5×OC=OCH_3_). ^31^P NMR (122 MHz, CDCl_3_) δ = -2.1. MALDI-TOF-MS (+) *m/z* 917.27 (M.Na^+^), calculated 917.23 for C_40_H_51_N_2_NaO_19_P^+^.

**(2R,3S,4R,5R,6S)-5-acetamido-6-(((2R,3S,4R,5R,6R)-5-acetamido-4-acetoxy-2-(acetoxymethyl)-6-(phosphonooxy)tetrahydro-2H-pyran-3-yl)oxy)-2-(acetoxymethyl)tetrahydro-2H-pyran-3,4-diyl diacetate (5)**

**4** (271 mg, 0.30 mmol, 1.00 eq) and 10% Pd/C (16.1 mg, 0.15 mmol, 0.50 eq) and 25 ml anhydrous MeOH were added to a dry flask, evacuated under vacuum and charged with an hydrogen atmosphere. The reaction mixture was then stirred at RT for 3 h under H_2_. The reaction mixture was filtered through celite and the solvent removed *in vacuo* to yield **5** (216 mg, 0.30 mmol, quantitative yield) as a colorless powder (DCM/MeOH 95:5, Rf = 0). ^1^H NMR (300 MHz, MeOD) δ = 5.48 (m, 1H, H-1a), 5.35 (t, *J* = 9.6 Hz, 1H, H-3a), 5.23 (t, *J* = 9.3 Hz, 1H, H-3b), 4.97 (d, *J* = 9.6 Hz, 1H, H-1b), 4.81 (d, *J* = 8.4 Hz, 1H, H-4b), 4.54 (d, *J* = 11.4 Hz, 1H, H-2a), 4.45 (dd, *J* = 12.3 Hz, 3.6 Hz, 1H, H-6a1), 4.23 (d, *J* = 10.5 Hz, 1H, H-6b1), 4.01-4.13 (m, 3H, H-6a2, H-6b2, H-2b), 3.91 (t, *J* = 9.3 Hz, 1H, H-5b), 3.81 (d, *J* = 9.0 Hz, 1H, H-4a), 3.59-3.66 (m, 1H, H-5a), 2.10, 2.06, 2.06, 1.99, 1.97, 1.94, 1.90 (7×s, 7×3H, 7×OAc). ^13^C NMR (75 MHz, MeOD) δ = 173.7, 173.7 (2×s, 2×NHC=OCH_3_), 172.5, 172.2, 172.2, 171.8, 171.3 (5×s, 5×OC=OCH_3_), 101.8 (s, C-1b), 95.8 (s, C-1a), 77.0 (s, C-4a), 73.6 (s, C-5a), 72.9 (s, C-3a), 72.3 (s, C-3b), 71.2 (s, C-5b), 70.0 (s, C-4b), 63.3 (s, C-6a), 63.1 (s, C-6b), 56.5 (s,C-2a), 53.3 (s, C-2b), 23.0, 22.5 (2×s, 2×NHC=OCH_3_), 21.1, 20.9, 20.7, 20.6, 20.6 (5×s, 5×OC=OCH_3_). ^31^P NMR (122 MHz, MeOD) δ = -1.7. ESI-MS (-) *m/z* 713.18 (M-[H^+^]), calculated 713.24 for C_26_H_38_N_2_O_19_P^-^.

**Ammonium (2E,6E)-3,7,11-trimethyldodeca-2,6,10-trien-1-yl phosphate (16b)**

CCl_3_CN (1.47 ml, 14.62 mmol, 6.50 eq) was quickly added to a stirred solution of **15b** (500 mg, 2.25 mmol, 1.00 eq) and Bu_4_NH_2_PO_4_ (1.48 g, 4.50 mmol, 2.00 eq) in 25 ml anhydrous DCM under Ar. The reaction mixture was stirred at RT for 20 min and the solvent was removed in high vacuum. The residue was then dissolved in 10 ml THF and 1 ml of a 25 % ammonium hydroxide solution in water was added. After 45 min, 25 ml of 1:1 toluene/MeOH was added and the reaction mixture was stirred for a further 20 min. After this time, the resultant precipitate was removed by filtration and solvents were removed *in vacuo*. The residue was washed with 3×50 ml petroleum ether and concentrated. A flash column chromatography on silica gel basified with a 25 % ammonium hydroxide solution in water (EtOAc/iPrOH/H_2_O 4:2:1, Rf = 0.39) and an ion exchange chromatography (Dowex 50WX8 in NH_4_Cl, elution in water) were made to purify the crude and get **16b** (432 mg, 1.28 mmol, 57 %) as a colorless lyophilisat after freeze-drying. ^1^H NMR (300 MHz, MeOH) δ = 5.40 (t, *J* = 6.9 Hz, 1H, H-2), 5.09-5.14 (m, 2H, H-6, H-10), 4.43 (t, *J* = 6.6 Hz, 2H, H-1), 1.97-2.12 (m, 8H, H-4, H-5, H-8, H-9), 1.69, 2×1.70, 1.60 (4×s, 4×3H, 4×CH_3_). ^13^C NMR (75 MHz, MeOD) δ = 140.9 (s, C-3), 136.3 (s, C-7), 132.1 (s, C-11), 125.8 (s, C-2), 125.5 (s, C-6), 125.2 (s, C-10), 63.4 (d, *J* = 4.4 Hz, C-1), 40.9 (s, C-4), 40.7 (s, C-8), 25.9 (s, C-5), 24.8 (s, C-9), 23.7 (s, C-15), 16.5 (s, C-14), 16.1 (s, C-12), 14.0 (s, C-13). ^31^P NMR (122 MHz, MeOD) δ = 1.2. ESI-MS (-) *m/z* 301.6 (M-[H^+^]), calculated 301.16 for C_15_H_26_O_4_P^-^.

**Ammonium (S)-3,7-dimethyloct-6-en-1-yl phosphate (16a)**

Following exactly the same procedure as for **16b** and starting from **15a** (500 mg, 3.20 mmol, 1.00 eq) and using CCl_3_CN (2.09ml, 20.80 mmol, 6.50 eq) and Bu_4_NH_2_PO_4_ (2.172 mg, 6.40 mmol, 2.00 eq) in 12.50 ml anhydrous DCM for 8 min, we got **16a** (342 mg, 1.27 mmol, 40 %) as a colorless lyophilisat after freeze-drying (EtOAc/iPrOH/H_2_O 4:2:1, Rf = 0.42). ^1^H NMR (300 MHz, MeOD) δ = 5.11 (tt, *J* = 6.9 Hz, 1.2 Hz, 1H, H-6), 3.86-3.94 (m, 2H, H-1), 1.95-2.05 (m, 2H, H-5), 1.63-1.73 (m, 5H, H-4, H-10), 1.6 (s, 3H, H-9), 1.31-1.48 (m, 2H, H-2), 1.13-1.23 (m, 1H, H-3), 0.92 (d, *J* = 6.6 Hz, 3H, H-8). ^13^C NMR (75 MHz, MeOD) δ = 131.9 (s, C-7), 126.0 (s, C-6), 64.7 (s, C-1), 39.1 (s, C-2), 38.5 (s, C-4), 30.5 (s, C-3), 26.6 (s, C-5), 26.1 (s, C-10), 20.0 (s, C-9), 18.0 (s, C-8). ^31^P NMR (122 MHz, MeOD) δ = 1.1. ESI-HRMS (-) *m/z* 235.1105 (M-[H^+^]), calculated 235.1103 for C_10_H_20_O_4_P^-^.

**(2R,3S,4R,5R,6S)-5-acetamido-6-(((2R,3S,4R,5R,6R)-5-acetamido-4-acetoxy-2-(acetoxymethyl)-6-((hydroxy((hydroxy(((2E,6E)-3,7,11-trimethyldodeca-2,6,10-trien-1-yl)oxy)phosphoryl)oxy)phosphoryl)oxy)tetrahydro-2H-pyran-3-yl)oxy)-2-(acetoxymethyl)tetrahydro-2H-pyran-3,4-diyl diacetate (17b)**

**16b** (278 mg, 0.83 mmol, 3.00 eq) and 1,1’-carbonyldiimidazole (671 mg, 4.14 mmol, 15.00 eq) were dissolved in 6 ml dry DMF and stirred at RT for 4 h under Ar. 1 ml anhydrous MeOH was added and the reaction mixture was stirred for an additional 2 h. A solution of **5** (197 mg, 0.28 mmol, 1.00 eq) in 5 ml dry DMF was added to the solution and stirred at RT for 6 days under Ar. The solvents were removed *in vacuo* and the crude was purified by flash column chromatography on silica gel basified with a 25 % ammonium hydroxide solution in water (EtOAc/iPrOH/H_2_O 4:2:1, Rf = 0.38) to yield **17b** (70 mg, 0.07 mmol, 25 %) as a colorless lyophilisat after freeze-drying. ^1^H NMR (300 MHz, MeOD) δ = 5.52 (dd, *J* = 7.2 Hz, 3 Hz, 1H, H-1a), 5.42 (m, 1H, H-2’), 5.35 (t, *J* = 9.6 Hz, 1H, H-3a), 5.21 (t, *J* = 9.3 Hz, 1H, H-3b), 5.07-5.15 (m, 2H, H-6’, H-10’), 4.97 (d, *J* = 9.9 Hz, 1H, H-1b), 4.82 (d, *J* = 8.7 Hz, 1H, H-4b), 4.60 (s, 1H, H-2a), 4.50-4.57 (m, 2H, H-1’), 4.43 (dd, *J* = 12.6 Hz, 3.9 Hz, 1H, H-6a1), 4.19-4.24 (m, 2H, H-6b1, H-6a2), 4.00-4.09 (m, 2H, H-6b2, H-2b), 3.94 (t, *J* = 9 Hz, 1H, H-5b), 3.78-3.83 (m, 1H, H-4a), 3.59-3.65 (m, 1H, H-5a), 2.11 (s, 3H, OAc), 1.97-2.03 (m, 23H, 5×3H + 8H, 5×OAc + H-4’, H-5’, H-8’, H-9’), 1.90 (s, 3H, OAc), 1.60-1.69 (m, 12H, H-12’, H-13’, H-14’, H-15’). ^13^C NMR (75 MHz, MeOD) δ = 174.0, 173.7 (2×s, 2×NHC=OCH_3_), 172.6, 172.2, 172.2, 171.8, 171.3 (5×s, 5×OC=OCH_3_), 141.3 (s, C-3’), 136.3 (s, C-7’), 132.1 (s, C-11’), 125.9 (s, C-2’), 125.4 (s, C-6’), 125.2 (s, C-10’), 101.8 (s, C-1b), 95.8 (s, C-1a), 76.9 (s, C-4a), 73.6 (s, C-5a), 73.1 (s, C-3a), 72.8 (s, C-3b), 71.0 (s, C-5b), 70.0 (s, C-4b), 64.0 (s, C-1’), 63.3 (s, C-6a), 63.1 (s, C-6b), 56.5 (s, C-2a), 53.4 (s, C-2b), 40.9 (s, C-4’), 40.7 (s, C-8’), 27.8 (s, C-5’), 25.9 (s, C-9’), 23.7 (s, C-15’), 23.0, 22.8 (2×s, 2×NHC=OCH_3_), 21.2, 21.0, 20.8, 20.6, 20.6 (5×s, 5×OC=OCH_3_), 17.8 (s, C-14’), 16.7 (s, C-12’), 16.1 (s, C-13’). ^31^P NMR (122 MHz, MeOD) δ = -10.2 (d, *J* = 19.4 Hz, P1), -13.1 (d, *J* = 19.4 Hz, P2). ESI-MS (-) *m/z* 997.34 (M-[H^+^]), calculated 997.33 for C_41_H_63_N_2_O_22_P_2_^-^. HRMS (-) *m/z* 997.3342 (M-[H^+^]), calculated 997.3356 for C_41_H_63_N_2_O_22_P_2_^-^.

***P*1-2-acetamido-O-(2-acetamido-2-deoxy-β-D-glucopyranosyl)-(1-4)-2-deoxy-α-D-glucopyranosyl *P*2-(2*E*,6*E*)-3,7,11-Trimethyldodeca-2,6,10-trien-1-yl diphosphate (1b)**

**17b** (40 mg, 0.04 mmol, 1.00 eq) was dissolved in 5 ml MeOH and an excess of a solution of ammonium hydroxide was added dropwise. The reaction mixture was stirred at RT for 12 h under Ar. MeOH was removed *in vacuo* and residues and water were removed in the freeze-dryer to yield **1b** (31.5 mg, 0.04 mmol, quantitative yield) as a colorless lyophilisat after freeze-drying (EtOAc/iPrOH/H_2_O 4:2:1, Rf = 0.08). ^1^H NMR (300 MHz, MeOD) δ = 5.57 (dd, *J* = 7.2 Hz, 3 Hz, 1H, H-1a), 5.42 (t, *J* = 5.4 Hz, 1H, H-2’), 5.09-5.15 (m, 2H, H-6’, H-10’), 4.56 (d, *J* = 8.4 Hz, 1H, H-2a), 4.51 (t, *J* = 6.3 Hz, 2H, H-1’), 3.34-4.01 (m, 12H, H-3a, H-3b, H-1b, H-4b, H-6a1, H-6b1, H-6a2, H-6b2, H-2b, H-5b, H-4a, H-5a), 1.99-2.11 (m, 14H, 2×3H + 8H, 2×NHC=OCH_3_ + H-4’, H-5’, H-8’, H-9’), 1.61-1.70 (m, 12H, H-12’, H-13’, H-14’, H-15’). ^13^C NMR (75 MHz, MeOD) δ = 174.2, 173.8 (2×s, 2×NHC=OCH_3_), 141.2 (s, C-3’), 136.3 (s, C-7’), 132.3 (s, C-11’), 125.9 (s, C-2’), 125.4 (s, C-6’), 125.2 (s, C-10’), 103.0 (s, C-1b), 95.8 (s, C-1a), 81.7 (s, C-4a), 78.2 (s, C-5a), 75.9 (s, C-3a), 73.3 (s, C-3b), 72.1 (s, C-5b), 71.2 (s, C-4b), 64.0 (s, C-1’), 62.6 (s, C-6a), 61.9 (s, C-6b), 57.6 (s, C-2a), 54.8 (s, C-2b), 40.9 (s, C-4’), 40.7 (s, C-8’), 27.6 (s, C-5’), 26.0 (s, C-9’), 23.7 (s, C-15’), 23.2, 22.1 (2×s, 2×NHC=OCH_3_), 17.8 (s, C-14’), 16.7 (s, C-12’), 16.1 (s, C-13’). ^31^P NMR (122 MHz, MeOD) δ = -10.4 (d, *J* = 19.4 Hz, P1), -12.7 (d, *J* = 19.4 Hz, P2). ESI-MS (-) *m/z* 787.28 (M-[H^+^]), calculated 787.28 for C_31_H_53_N_2_O_17_P_2_^-^. HRMS (-) *m/z* 787.2825 (M-[H^+^]), calculated 787.2825 for C_41_H_63_N_2_O_22_P_2_^-^.

**(2R,3S,4R,5R,6S)-5-acetamido-6-(((2R,3S,4R,5R,6R)-5-acetamido-4-acetoxy-2-(acetoxymethyl)-6-(((((((S)-3,7-dimethyloct-6-en-1-yl)oxy)(hydroxy)phosphoryl)oxy)(hydroxy)phosphoryl)oxy)tetrahydro-2H-pyran-3-yl)oxy)-2-(acetoxymethyl)tetrahydro-2H-pyran-3,4-diyl diacetate (17a)**

Following exactly the same procedure as for **17b** and starting from **16a** (60 mg, 0.22 mmol, 1.00 eq) and using 1,1’-carbonyldiimidazole (180 mg, 1.11 mmol, 5.00 eq) and **5** (475.8 mg, 0.67 mmol, 3.00 eq), we obtained **17a** (83 mg, 0.09 mmol, 39 %) as a colorless lyophilisat after freeze-drying (EtOAc/iPrOH/H_2_O 4:2:1, Rf = 0.30). ^1^H NMR (300 MHz, MeOD) δ = 5.51 (dd, *J* = 7.2 Hz, 3.0 Hz, 1H, H-1a), 5.33 (t, *J* = 9.6 Hz, 1H, H-3a), 5.08-5.17 (m, 3H, H-3b, H-6’, NHaAc), 4.92-4.99 (m, 2H, H-1b, NHbAc), 4.79 (d, *J* = 8.4 Hz, 1H, H-4b), 4.57 (dd, *J* = 12.3 Hz, 1.5 Hz, 1H, H-2a), 4.43 (dd, *J* = 12.3 Hz, 3.9 Hz, 2H, H-1’), 4.14-4.22 (m, 2H, H-6a1, H-6b1), 4.00-4.07 (m, 3H, H-6a2, H-6b2, H-2b), 3.89 (t, *J* = 9.3 Hz, 1H, H-5b), 3.78-3.82 (dd, *J* = 10.2 Hz, 1.5 Hz, 1H, H-4a), 3.65 (dd, *J* = 10.5 Hz, 8.7 Hz, 1H, H-5a), 2.10, 2.05, 2.02 (3×s, 3×3H, 3×OAc), 1.97-1.98 (br s, 11H, 3×3H + 2H, 2×NHAc + OAc + H-5’), 1.90 (s, 3H, OAc), 1.68-1.75 (m, 1H, H-4’a), 1.66, 1.59 (2×s, 2×3H, H-9’, H-10’), 1.28-1.46 (m, 3H, H-4’b, H-2’), 1.12-1.20 (m, 1H, H-3’), 0.90 (d, *J* = 6.6 Hz, 3H, H-8’). ^13^C NMR (75 MHz, MeOD) δ = 173.9, 173.6 (2×s, 2×NHC=OCH_3_), 172.5, 172.2, 172.1, 171.8, 171.3 (5×s, 5×OC=OCH_3_), 135.5 (s, C-7’), 126.0 (s, C-6’), 101.9 (s, C-1b), 95.7 (s, C-1a), 77.0 (s, C-4a), 73.7 (s, C-5a), 73.2 (s, C-3a), 72.8 (s, C-3b), 71.0 (s, C-5b), 70.0 (s, C-4b), 65.8 (s, C-1’), 65.7 (s, C-2’), 63.2 (s, C-6a), 63.1 (s, C-6b), 56.4 (s, C-2a), 53.4 (s, C-2b), 38.8 (s, C-4’), 30.5 (s, C-3’), 26.5 (s, C-5’), 26.0 (s, C-10’), 23.0, 22.9 (2×s, 2×NHC=OCH_3_), 21.2, 21.0, 20.8, 20.7, 20.6 (5×s, 5×OC=OCH_3_), 19.8 (s, C-9’), 17.8 (s, C-8’). ^31^P NMR (122 MHz, MeOD) δ = -10.1 (d, *J* = 20.8 Hz, P1), -13.1 (d, *J* = 20.8 Hz, P2). ESI-HRMS (-) *m/z* 931.2884 (M-[H^+^]), calculated 931.2873 for C_36_H_57_N_2_O_22_P_2_^-^.

***P*1-2-acetamido-O-(2-acetamido-2-deoxy-β-D-glucopyranosyl)-(1-4)-2-deoxy-α-D-glucopyranosyl *P*2-(S)-3,7-dimethyloct-6-en-1-yl diphosphate (1a)**

Following exactly the same procedure as for **1b** and starting from **17a** (73 mg, 0.08 mmol, 1.00 eq), we obtained after 12 h **1a** (57 mg, 0.08 mmol, quantitative yield) as a colorless lyophilisat after freeze-drying (EtOAc/iPrOH/H_2_O 4:2:1, Rf = 0.02). ^1^H NMR (300 MHz, MeOD) δ = 5.48 (dd, *J* = 7.2 Hz, 2.7 Hz, 1H, H-1a), 5.07 (d, *J* = 3.0 Hz, 1H, H-6’), 4.51 (d, *J* = 8.1 Hz, 1H, H-2a), 3.94-4.00 (m, 3H, H-1’, H-3a), 3.85-3.89 (m, 2H, H-3b, H-1b), 3.70-3.77 (m, 2H, H-4b, H-6a1), 3.63-3.67 (m, 2H, H-6b1, H-6a2), 3.56-3.60 (m, 1H, H-6b2), 3.50-3.53 (m, 1H, H-2b), 3.45-3.49 (m, 1H, H-5b), 3.32-3.38 (m, 1H, H-4a), 3.26-3.28 (m, 1H, H-5a), 1.99, 1.98 (2×s, 2×3H, 2×NHAc), 1.93 (s, 2H, H-6’), 1.64-1.68 (m, 1H, H-4’a), 1.62, 1.65 (2×s, 2×3H, H-9’, H-10’), 1.24-1.44 (m, 3H, H-2’, H-4’b), 1.10-1.17 (m, 1H, H-3’), 0.86 (d, *J* = 6.6 Hz, 3H, H-8’). ^13^C NMR (75 MHz, MeOD) δ = 174.2, 173.8 (2×s, 2×NHC=OCH_3_), 135.9 (s, C-7’), 125.9 (s, C-6’), 103.1 (s, C-1b), 95.9 (s, C-1a), 81.6 (s, C-4a), 78.2 (s, C-5a), 75.8 (s, C-3a), 73.3 (s, C-3b), 72.1 (s, C-5b), 71.5 (s, C-4b), 65.7 (s, C-1’), 65.6 (s, C-2’), 62.6 (s, C-6a), 61.7 (s, C-6b), 57.5 (s, C-2a), 54.8 (s, C-2b), 38.4 (s, C-4’), 30.5 (s, C-3’), 26.5 (s, C-5’), 26.0 (s, C-10’), 23.3, 23.1 (2×s, 2×NHC=OCH_3_), 19.9 (s, C-9’), 17.8 (s, C-8’). ^31^P NMR (122 MHz, MeOD) δ = -10.3 (d, *J* = 20.8 Hz, P1), -12.7 (d, *J* = 19.3 Hz, P2). ESI-HRMS (-) *m/z* 721.2355 (M-[H^+^]), calculated 721.2346 for C_26_H_47_N_2_O_17_P_2_^-^.

**(S)-tert-butyl((3,7-dimethyloct-6-en-1-yl)oxy)diphenylsilane (7)**

**6** (10.00 g, 64.00 mmol, 1.00 eq) and imidazole (4.79 g, 70.40 mmol, 1.10 eq) were added to a dried flask and dissolved in 20 ml DMF. TBDPSCl (18.49 g, 67.19 mmol, 1.05 eq) was added and the mixture was stirred at RT for 16 h. 250 ml EtOAc were added to dissolved the reaction mixture which was washed with 250 ml 0.5 M HCl, saturated NaHCO_3_, water and brine respectively and concentrated *in vacuo* to yield **7** (25.26 g, 64.00 mmol) as a colorless oil which was used in the following step without further purification (Hexane, Rf = 0.19). ^1^H NMR (300 MHz, CDCl_3_) δ = 7.66-7.69 (m, 4H, 4×Si-Ph-*o*-H), 7.38-7.42 (m, 6H, 4×Si-Ph-*m*-H, 2×Si-Ph-*p*-H), 5.09 (tt, *J* = 7.2 Hz, 2.7 Hz, 1.5 Hz, 1H, H-6), 3.67-3.72 (m, 2H, H-1), 1.94-1.98 (m, 2H, H-5), 1.68, 1.60 (2×s, 2×3H, H-9, H-10), 1.25-1.40 (m, 2H, H-2), 1.09-1.19 (m, 2H, H-4), 1.08 (s, 1H, H-3), 1.05 (s, 9H, C(CH_3_)_3_), 0.83 (d, *J* = 6.6 Hz, 3H, H-8). ^13^C NMR (75 MHz, CDCl_3_) δ = 135.6 (s, 4×Si-Ph-*o*-C), 134.8 (s, C-7), 134.2 (s, C-6), 131.0 (s, 2×Si-Ph-C), 129.5 (s, 4×Si-Ph-*m*-C), 127.6 (s, 2×Si-Ph-*p*-C), 62.2 (s, C-1), 39.7 (s, C-2), 37.2 (s, C-4), 29.1 (s, C-3), 26.9 (s, C(CH_3_)_3_), 25.7 (s, C-5), 25.5 (s, C-8), 19.6 (s, C-9), 19.2 (s, C-10), 17.7 (s, C(CH_3_)_3_). ESI MS (+) *m/z* 395.28 (M+[H^+^]), calculated 395.28 for C_26_H_39_OSi^+^.

**(S)-6-((tert-butyldiphenylsilyl)oxy)-4-methylhexanal (8)**

A steady stream of ozone generated with an ozonizer was bubbled through a solution of **7** (25.00 g, 63.35 mmol, 1.00 eq) in 150 ml of a mixture DCM/MeOH 5:1 for 1 h at -78°C. When the color of the reaction mixture turned blue, meaning the reaction is complete, a steady stream of oxygen was bubbled through the reaction mixture to remove the excess of ozone for 30 min. Under an Ar atmosphere, dimethyl sulfide (23.26 ml, 316.75 mmol, 5.00 eq) was added to the solution at -78°C which was allowed to warm up to RT overnight. Volatile materials were removed under reduced pressure and the residue was diluted in 200 ml water and extracted with 2×200 ml hexane. The combined organic fractions were washed with 400 ml 5% HCl, brine, dried over Na_2_SO_4_, filtered and concentrated. A flash column chromatography on silica gel (EtOAc/hexane 3:97, Rf = 0.21) was made to purify the crude and yield **8** (21.50 g, 58.33 mmol, 92 %) as a colorless oil. ^1^H NMR (300 MHz, CDCl_3_) δ = 9.75 (t, *J* = 1.8 Hz, 1H, H-6), 7.65-7.68 (m, 4H, 4×Si-Ph-*o*-H), 7.38-7.42 (m, 6H, 4×Si-Ph-*m*-H, 2×Si-Ph-*p*-H), 3.67-3.73 (m, 2H, H-1), 3.31 (d, *J* = 1.2 Hz, 2H, H-5), 2.35-2.42 (m, 2H, H-4), 1.59-1.64 (m, 2H, H-2), 1.12 (s, 1H, H-3), 1.05 (s, 9H, C(CH_3_)_3_), 0.85 (d, *J* = 6.3 Hz, 3H, H-7). ^13^C NMR (75 MHz, CDCl_3_) δ = 202.7 (s, C-6), 135.6 (s, 4×Si-Ph-*o*-C), 134.0 (s, 2×Si-Ph-C), 129.6 (s, 4×Si-Ph-*m*-C), 127.6 (s, 2×Si-Ph-*p*-C), 61.9 (s, C-1), 52.6 (s, C-5), 41.6 (s, C-2), 39.2 (s, C-4), 29.2 (s, C-3), 28.9 (s, C(CH_3_)_3_), 26.9 (s, C(CH_3_)_3_), 19.4 (s, C-7). ESI MS (+) *m/z* 391.21 (M.Na^+^), calculated 391.21 for C_23_H_32_NaO_2_Si^+^.

**(S,Z)-8-((tert-butyldiphenylsilyl)oxy)-2,6-dimethyloct-2-en-1-ol (9)**

To a stirred solution of ethyltriphenylphosphonium bromide (22.16 g, 59.69 mmol, 1.10 eq) in 180 ml THF was added n-BuLi (22.00 ml, 59.69 mmol, 1.10 eq, 2.70 M in heptan) dropwise at 0°C. After 1 h at this temperature, the reaction mixture was cooled down to -78°C and **8** (20.00 g, 54.26 mmol, 1.00 eq) was added dropwise and the mixture was stirred for 5 min at -78°C to get a red solution. Additional n-BuLi (22.00 ml, 59.69 mmol, 1.10 eq, 2.70 M in heptan) was added dropwise, resulting in the formation of the deep red ylide. The reaction mixture was allowed to reach 0°C and paraformaldehyde (3.26 g, 108.52 mmol, 2.00 eq) was added. The reaction mixture was stirred for 1 h at 0°C and overnight at RT. Ice water was added and the mixture was extracted with Et_2_O. The organic phase was dried over Na_2_SO_4_, filtered and concentrated *in vacuo*. The residue was purified by flash column chromatography on silica gel (pet ether/ether 7:1 to 5:1 to 3:1, Rf = 0.21 in 3:1) to yield **9** (8.17 g, 19.89 mmol, 37 %) as a colorless oil. ^1^H NMR (300 MHz, CDCl_3_) δ = 7.65-7.68 (m, 4H, 4×Si-Ph-*o*-H), 7.38-7.41 (m, 6H, 4×Si-Ph-*m*-H, 2×Si-Ph-*p*-H), 5.27 (t, *J* = 7.2 Hz, 1H, H-6), 4.12 (s, 2H, H-1), 3.68-3.70 (m, 2H, H-8), 1.98-2.08 (m, 2H, H-5), 1.78 (s, 3H, H-10), 1.57-1.63 (m, 2H, H-4), 1.29-1.35 (m, 2H, H-2), 1.11 (s, 1H, H-3), 1.04 (s, 9H, C(CH_3_)_3_), 0.82 (d, *J* = 6.6 Hz, 3H, H-9). ^13^C NMR (75 MHz, CDCl_3_) δ = 135.6 (s, 4×Si-Ph-*o*-C), 134.2 (s, C-7), 134.1 (s, 2×Si-Ph-C), 129.6 (s, 2×Si-Ph-*p*-C), 128.8 (s, 4×Si-Ph-*m*-C), 127.6 (s, C-6), 62.1 (s, C-8), 61.7 (s, C-1), 39.5 (s, C-2), 37.4 (s, C-4), 29.0 (s, C-3), 26.9 (s, C(CH_3_)_3_), 25.1 (s, C-5), 21.3 (s, C-9), 19.6 (s, C-10), 19.3 (s, C(CH_3_)_3_). No mass could be observed by ESI-MS (+/-).

**(S,Z)-tert-butyl((8-chloro-3,7-dimethyloct-6-en-1-yl)oxy)diphenylsilane (10)**

MsCl (7.19 g, 62.75 mmol, 3.00 eq) was added dropwise to a stirred solution of **9** (8.59 g, 20.92 mmol, 1.00 eq), LiCl (2.66 g, 62.75 mmol, 3.00 eq) and 2,4,6-collidine (10.14 g, 83.67 mmol, 4.00 eq) in 100 ml DMF at -3°C. The reaction mixture was stirred at this temperature for 2 h, was then poured onto 250 ml ice cold saturated NaHCO_3_ and extracted with 3×250 ml of a mixture hexane/diethyl ether 1:1. The combined organic fractions were washed with 750 ml saturated NH_4_Cl and brine respectively, dried over Na_2_SO_4_, filtered and concentrated *in vacuo*. A flash column chromatography on silica gel (EtOAc/pet ether 5:95, Rf = 0.82) was made to purify the crude and yield **10** (6.07 g, 14.15 mmol, 68%) as a yellow oil. ^1^H NMR (300 MHz, CDCl_3_) δ = 7.67-7.70 (m, 4H, 4×Si-Ph-*o*-H), 7.37-7.44 (m, 6H, 4×Si-Ph-*m*-H, 2×Si-Ph-*p*-H), 5.37 (dt, *J* = 7.5 Hz, 1.5 Hz, 1H, H-6), 4.06 (s, 2H, H-1), 3.67-3.75 (m, 2H, H-8), 2.02-2.11 (m, 2H, H-5), 1.82 (s, 3H, H-10), 1.59-1.66 (m, 2H, H-4), 1.32-1.42 (m, 2H, H-2), 1.17-1.22 (m, 1H, H-3), 1.05 (s, 9H, C(CH_3_)_3_), 0.86 (d, *J* = 6.6 Hz, 3H, H-9). ^13^C NMR (75 MHz, CDCl_3_) δ = 135.6 (s, 4×Si-Ph-*o*-C), 134.2 (s, C-7), 131.6 (s, C-6), 131.1 (s, 2×Si-Ph-C), 129.5 (s, 2×Si-Ph-*p*-C), 127.6 (s, 4×Si-Ph-*m*-C), 62.1 (s, C-1), 43.8 (s, C-8), 39.5 (s, C-2), 36.9 (s, C-4), 29.1 (s, C-3), 26.9 (s, C(CH_3_)_3_), 25.4 (s, C-5), 21.6 (s, C-9), 19.5 (s, C-10), 19.2 (s, C(CH_3_)_3_). No mass could be observed by ESI-MS (+/-).

**(Z)-1-bromo-3,7-dimethylocta-2,6-diene (12c)**

**11c** (1.76 g, 11.42 mmol, 1.00 eq) was dissolved in 4 ml THF and PBr_3_ (1.54 g, 5.71 mmol, 0.50 eq) was added dropwise at 0°C. The reaction was stirred at this temperature for 1 h, was then diluted in 4 ml of a mixture diethyl ether/water 1:1, washed with 4 ml saturated NaHCO_3_, water and brine respectively, dried over Na_2_SO_4_, filtered and concentrated *in vacuo* to yield **12c** (2.20 g, 10.13 mmol) as a yellow oil which was used in the next step without further purification**.** ^1^H NMR (300 MHz, CDCl_3_) δ = 5.53 (t, *J* = 8.7 Hz, 1H, H-2), 5.10-5.14 (m, 1H, H-6), 4.00 (d, *J* = 8.7 Hz, 2H, H-1), 2.16 (s, 4H, H-4, H-5), 1.77, 1.69, 1.61 (3×s, 3×3H, 3×CH_3_). ^13^C NMR (75 MHz, CDCl_3_) δ = 143.4 (s, C-3), 132.4 (s, C-7), 123.5 (s, C-6), 121.4 (s, C-2), 31.8 (s, C-4), 29.4 (s, C-1), 26.3 (s, C-5), 25.7 (s, C-10), 23.6 (s, C-9), 17.7 (s, C-8). No mass could be observed by ESI-MS (+/-).

**(Z)-(3,7-dimethylocta-2,6-dienylsulfonyl)benzene (13c)**

**12c** (2.00g, 9.21 mmol, 1.00 eq) was added to a solution of PhSO_2_Na (2.27 g, 13.82 mmol, 1.50 eq) in 40 ml DMF at 0°C and was stirred at this temperature for 4 h. The reaction mixture was diluted in 40 ml EtOAc, washed with 100 ml 1M HCl, saturated NaHCO_3_, water and brine respectively, dried over Na_2_SO_4_, filtered and concentrated *in vacuo*. The residue was purified by flash column chromatography on silica gel (EtOAc/pet ether 5:95, Rf = 0.09) to yield **13c** (1.80g, 6.47 mmol, 63% over 2 steps) as a yellow oil. ^1^H NMR (300 MHz, CDCl_3_) δ = 7.84-7.87 (m, 2H, 2×SO_2_Ph-*o*-H), 7.60-7.65 (m, 1H, SO_2_Ph-*p*-H), 7.50-7.55 (m, 2H, 2×SO_2_Ph-*m*-H), 5.19 (dt, *J* = 7.5 Hz, 0.6 Hz, 1H, H-2), 4.90-4.96 (m, 1H, H-6), 3.79 (dd, *J* = 7.8 Hz, 0.6 Hz, 2H, H-1), 1.74-1.85 (m, 4H, H-4, H-5), 1.71, 1.63, 1.53 (3×s, 3×3H, 3×CH_3_). ^13^C NMR (75 MHz, CDCl_3_) δ = 146.1 (s, C-3), 138.9 (s, SO_2_Ph-C), 133.5 (s, SO_2_Ph-*p*-C), 132.3 (s, C-7), 129.0 (s, 2×SO_2_Ph-*o*-C), 128.4 (s, 2×SO_2_Ph-*m*-C), 123.3 (s, C-6), 110.9 (s, C-2), 56.0 (s, C-1), 31.9 (s, C-4), 26.0 (s, C-5), 25.6 (s, C-10), 23.5 (s, C-9), 17.7 (s, C-8). ESI-MS (+) *m/z* 301.12 (M.Na^+^), calculated 301.12 for C_16_H_22_NaO_2_S^+^.

**(2E,6E)-1-bromo-3,7,11-trimethyldodeca-2,6,10-triene (12d)**

Following exactly the same procedure as for **12c** and starting from **11d** (2.00 g, 8.99 mmol, 1.00 eq) and using PBr_3_ (0.43 ml, 4.50 mmol, 0.50 eq) in 5 ml anhydrous THF, we got **12d** (1.95 mg, 6.84 mmol) as a yellow oil which was used in the next step without further purification. ^1^H NMR (300 MHz, CDCl_3_) δ = 5.53 (dt, *J* = 8.4 Hz, 1.2 Hz, 1H, H-2), 5.06-5.10 (m, 2H, H-6, H-10), 4.02 (d, *J* = 8.7 Hz, 2H, H-1), 1.97-2.10 (m, 8H, H-4, H-5, H-8, H-9), 1.73, 1.68, 2×1.59 (4×s, 4×3H, 4×CH_3_). ^13^C NMR (75 MHz, CDCl_3_) δ = 143.6 (s, C-3), 135.6 (s, C-7), 131.3 (s, C-11), 124.3 (s, C-10), 123.4 (s, C-6), 120.6 (s, C-2), 39.7 (s, C-8), 39.5 (s, C-4), 29.6 (s, C-1), 26.7 (s, C-9), 26.1 (s, C-5), 25.7 (s, C-15), 17.7 (s, C-14), 16.1 (s, C-13), 16.0 (s, C-12). ESI MS (+) *m/z* 307.10 (M.Na^+^), calculated 307.14 for C_15_H_25_BrNa^+^.

**(((2E,6E)-3,7,11-trimethyldodeca-2,6,10-trien-1-yl)sulfonyl)benzene (13d)**

Following exactly the same procedure as for **13c** and starting from **12d** (1.95 g, 6.84 mmol, 1.00 eq) and using PhSO_2_Na (1.69 g, 10.26 mmol, 1.50 eq) in 40 ml anhydrous DMF, we got **13d** (1.76 mg, 5.09 mmol, 56 % over 2 steps) as a yellow oil after purification (Rf = 0.11, pet ether/EtOAc 95:5). ^1^H NMR (300 MHz, CDCl_3_) δ = 7.84-7.87 (m, 2H, 2×SO_2_Ph-*o*-H), 7.60-7.65 (m, 1H, SO_2_Ph-*p*-H), 7.49-7.54 (m, 2H, 2×SO_2_Ph-*m*-H), 5.18 (t, *J* = 7.8 Hz, 1H, H-2), 5.04-5.09 (m, 2H, H-6, H-10), 3.80 (d, *J* = 7.8 Hz, 2H, H-1), 1.94-2.06 (m, 8H, H-4, H-5, H-8, H-9), 1.67, 1.59, 1.57, 1.30 (4×s, 4×3H, 4×CH_3_). ^13^C NMR (75 MHz, CDCl_3_) δ = 146.4 (s, C-3), 138.8 (s, SO_2_Ph-C), 135.7 (s, C-7), 133.5 (s, SO_2_Ph-*p*-C), 131.4 (s, C-11), 128.9 (s, 2×SO_2_Ph-*o*-C), 128.6 (s, 2×SO_2_Ph-*m*-C), 124.2 (s, C-10), 123.3 (s, C-6), 110.9 (s, C-3), 56.1 (s, C-1), 39.7 (s, C-4), 26.7 (s, C-8), 26.2 (s, C-5), 25.7 (s, C-9), 17.7 (s, C-15), 16.2 (s, C-14), 2×16.0 (2×s, C-13, C-12). ESI-MS (+) *m/z* 369.19 (M.Na^+^), calculated 369.18 for C_21_H_30_NaO_2_S^+^.

**Tert-butyldiphenyl(((3S,6Z,10Z)-3,7,11,15-tetramethyl-9-(phenylsulfonyl)hexadeca-6,10,14-trien-1-yl)oxy)silane (14c)**

n-BuLi (1.45 ml, 3.90 mmol, 1.00 eq, 2.70 M in heptan) was added slowly to a stirred solution of **13c** (1.09 g, 3.90 mmol, 1.00 eq) and DMPU (2.17 ml, 17.94 mmol, 4.60 eq) in 6.5 ml THF at -78°C. After stirring 2 h at this temperature, a solution of **10** (2.01 g, 4.68 mmol, 1.20 eq) in 6.5 ml THF was added dropwise over 90 min. The reaction mixture was allowed to warm up to -30°C over 2 h and stirred at this temperature for a further 2 h. The reaction was quenched by pouring onto 60 ml saturated NH_4_Cl. The aqueous phase was extracted with 3×150 ml of a mixture hexane/diethyl ether 1:1 and the combined organic fractions were washed with 3×450 ml brine, dried over Na_2_SO_4_, filtered and concentrated *in vacuo*. The residue was purified by flash column chromatography on silica gel (DCM/pet ether 1:2 to 1:1, Rf = 0.30 in DCM/pet ether 1:1) to yield **14c** (1.95 g, 2.91 mmol, 90 %) as a colorless oil after purification as well as (S,Z)-tert-butyl((8-chloro-3,7-dimethyloct-6-en-1-yl)oxy)diphenylsilane (627 mg, 1.46 mmol). ^1^H NMR (300 MHz, CDCl_3_) δ = 7.83-7.85 (m, 2H, 2×SO_2_Ph-*o*-H), 7.65-7.69 (m, 4H, 4×Si-Ph-*o*-H), 7.57-7.59 (m, 1H, SO_2_Ph-*p*-H), 7.50-7.52 (m, 2H, 2×SO_2_Ph-*m*-H), 7.35-7.42 (m, 6H, 4×Si-Ph-*m*-H, 2×Si-Ph-*p*-H), 5.17 (t, *J* = 7.2 Hz, 1H, H-6), 4.98 (d, *J* = 10.8 Hz, 1H, H-10), 4.88 (t, *J* = 7.2 Hz, 1H, H-14), 3.81-3.86 (m, 1H, H-9), 3.65-3.70 (m, 2H, H-1), 2.69-2.73 (m, 1H, H-8a), 2.48-2.53 (m, 1H, H-8b), 1.85-1.93 (m, 2H, H-5), 1.67-1.78 (m, 4H, H-12, H-13), 1.64, 1.63, 1.61 (3×s, 3×3H, H-17, H-19, H-20), 1.57-1.59 (m, 2H, H-4), 1.52 (s, 3H, H-18), 1.43-1.46 (m, 1H, H-3), 1.26-1.31 (m, 2H, H-2), 1.05 (s, 9H, C(CH_3_)_3_), 0.80 (d, *J* = 6.0 Hz, 3H, H-16). ^13^C NMR (75 MHz, CDCl_3_) δ = 145.2 (s, C-11), 138.1 (s, SO_2_Ph-C), 135.6 (s, 4×Si-Ph-*o*-C), 134.2 (s, 2×Si-Ph-C), 133.4 (s, SO_2_Ph-*p*-C), 132.0 (s, C-15), 129.6 (s, C-7), 129.5 (s, 2×Si-Ph-*p*-C), 129.3 (s, 2×SO_2_Ph-*o*-C), 129.2 (s, 2× SO_2_Ph-*m*-C), 128.7 (s, C-6), 127.6 (s, 4×Si-Ph-*m*-C), 123.5 (s, C-14), 117.6 (s, C-10), 63.4 (s, C-9), 62.2 (s, C-1), 39.7 (s, C-2), 37.2 (s, C-4), 32.0 (s, C-12), 30.1 (s, C-8), 29.6 (s, C-3), 26.9 (s, C(CH_3_)_3_), 25.6 (s, C-5), 25.5 (s, C-13), 23.8 (s, C-20), 23.3 (s, C-18), 19.5 (s, C-17), 19.2 (s, C-19), 17.7 (s, C(CH_3_)_3_), 17.7 (s, C-16). ESI-MS (+) *m/z* 693.38 (M.Na^+^), calculated 693.38 for C_42_H_58_NaO_3_SSi^+^.

**(3S,6Z,10Z)-3,7,11,15-tetramethylhexadeca-6,10,14-trien-1-ol (15c)**

**14c** (1.94 g, 2.89 mmol, 1.00 eq) was dissolved in 40 ml THF and the solution was cooled down to 0°C. TBAF (4.53 ml, 5.78 mmol, 2.00 eq, 1.00 M in THF) was added and the reaction mixture was stirred at 0°C for 15 h, was then diluted with 400 ml diethyl ether, washed with 3×400 ml brine. The organic layer was dried over Na_2_SO_4_ and concentrated *in vacuo*. The residue was dissolved in 60 ml THF and (dppp)PdCl_2_ (563 mg, 0.95 mmol, 0.33 eq) was added. At 0°C, LiEt_3_BH (14.50 ml, 14.46 mmol, 5.00 eq, 1.00 M in THF) was added dropwise and the mixture was stirred at this temperature for 2 h. 80 ml saturated NH_4_Cl wan then added and the reaction mixture was extracted with 3×120 ml diethyl ether. The organic layers were washed with 3×400 ml brine, dried over Na_2_SO_4_, filtered and concentrated *in vacuo*. The residue was purified by flash column chromatography on silica gel (DCM, Rf = 0.27) to yield **15c** (703 mg, 2.40 mmol, 83 %) as a colorless oil after purification. ^1^H NMR (300 MHz, CDCl_3_) δ = 5.09-5.13 (m, 3H, H-6, H-10, H-14), 3.64-3.70 (m, 2H, H-1), 1.96-2.03 (m, 10H, H-5, H-8, H-9, H-12, H-13), 1.68 (s, 9H, H-18, H-19, H-20), 1.61 (s, 3H, H-17), 1.54-1.58 (m, 2H, H-4), 1.33-1.43 (m, 2H, H-2), 1.31 (s, 1H, H-3), 0.90 (d, *J* = 6.6 Hz, 3H, H-16). ^13^C NMR (75 MHz, CDCl_3_) δ = 135.3 (s, C-7), 135.0 (s, C-11), 131.5 (s, C-15), 125.4 (s, C-6), 125.1 (s, C-10), 124.4 (s, C-14), 61.2 (s, C-1), 40.0 (s, C-2), 37.5 (s, C-4), 32.3 (s, C-8), 32.0 (s, C-3), 29.3 (s, C-12), 26.7 (s, C-13), 26.4 (s, C-5), 25.7 (s, C-9), 25.3 (s, C-20), 23.4 (s, C-17), 23.4 (s, C-18), 19.5 (s, C-19), 17.6 (s, C-16). ESI-MS (+) *m/z* 293.28 (M+[H^+^]), calculated 293.28 for C_20_H_37_O^+^.

**Ammonium (S,6Z,10Z)-3,7,11,15-tetramethylhexadeca-6,10,14-trien-1-yl phosphate (16c)**

Following exactly the same procedure as for **16b** and starting from **15c** (690 mg, 2.36 mmol, 1.00 eq) and using CCl_3_CN (1.54 ml, 15.33 mmol, 6.50 eq) and Bu_4_NH_2_PO_4_ (1.60 mg, 4.72 mmol, 2.00 eq) in 15 ml anhydrous DCM for 8 min, we got **16c** (356 mg, 0.88 mmol, 37 %) as a yellow lyophilisat after freeze-drying (EtOAc/iPrOH/H_2_O 4:2:1, Rf = 0.37). ^1^H NMR (300 MHz, MeOD) δ = 5.13 (m, 3H, H-6, H-10, H-14), 3.86-3.94 (m, 2H, H-1), 1.98-2.05 (m, 10H, H-5, H-8, H-9, H-12, H-13), 1.64-1.72 (m, 11H, H-4, H-18, H-19, H-20), 1.61 (s, 3H, H-17), 1.36-1.45 (m, 2H, H-2), 1.13-1.23 (m, 1H, H-3), 0.92 (d, *J* = 6.6 Hz, 3H, H-16). ^13^C NMR (75 MHz, MeOD) δ = 136.3 (s, C-7), 135.8 (s, C-11), 132.4 (s, C-15), 126.7 (s, C-6), 126.2 (s, C-10), 125.4 (s, C-14), 64.5 (s, C-1), 39.1 (s, C-2), 38.8 (s, C-4), 33.2 (s, C-8), 32.9 (s, C-3), 30.6 (s, C-12), 27.8 (s, C-13), 27.6 (s, C-5), 26.4 (s, C-9), 26.0 (s, C-20), 23.8 (s, C-17), 23.7 (s, C-18), 19.8 (s, C-19), 17.8 (s, C-16). ^31^P NMR (122 MHz, MeOD) δ = 1.4. ESI-HRMS (-) *m/z* 371.2357 (M-[H^+^]), calculated 371.2366 for C_20_H_36_O_4_P^-^.

**Tert-butyl(((3S,6Z,10E,14E)-3,7,11,15,19-pentamethyl-9-(phenylsulfonyl)icosa-6,10,14,18-tetraen-1-yl)oxy)diphenylsilane (14d)**

Following exactly the same procedure as for **14c** and starting from **13d** (1.377 g, 3.97 mmol, 1.00 eq) and **10** (2.046 g, 4.77 mmol, 1.20 eq) and using DMPU (2.20 ml, 18.28 mmol, 4.60 eq) and n-BuLi (1.47 ml, 3.97 mmol, 1.00 eq, 2.70 M in heptan) in 16 ml anhydrous THF, we got **14d** (2.071 g, 2.80 mmol, 85 %) as a colorless oil after purification (Rf = 0.24, pet ether/DCM 1:1) as well as (S,Z)-tert-butyl((8-chloro-3,7-dimethyloct-6-en-1-yl)oxy)diphenylsilane (635 mg, 1.48 mmol). ^1^H NMR (300 MHz, CDCl_3_) δ = 7.84-7.87 (m, 2H, 2×SO_2_Ph-*o*-H), 7.66-7.69 (m, 4H, 4×Si-Ph-*o*-H), 7.57-7.60 (m, 1H, SO_2_Ph-*p*-H), 7.47-7.52 (m, 2H, 2×SO_2_Ph-*m*-H), 7.36-7.42 (m, 6H, 4×Si-Ph-*m*-H, 2×Si-Ph-*p*-H), 5.16 (t, *J* = 7.2 Hz, 1H, H-6), 5.03-5.08 (m, 2H, H-14, H-18), 4.95 (d, *J* = 10.5 Hz, 1H, H-10), 3.83-3.91 (m, 1H, H-9), 3.65-3.70 (m, 2H, H-1), 2.72-2.79 (m, 1H, H-8a), 2.47-2.58 (m, 1H, H-8b), 1.92-2.07 (m, 10H, H-5, H-12, H-13, H-16, H-17), 1.67 (s, 3H-25), 1.55-1.59 (m, 11H, H-4, H-24, H-23, H-22), 1.25-1.34 (m, 3H, H-2, H-3), 1.17 (s, 3H, H-21), 1.05 (s, 9H, C(CH_3_)_3_), 0.81 (dd, *J* = 6.3 Hz, 1.5 Hz, 3H, H-20). ^13^C NMR (75 MHz, CDCl_3_) δ = 145.2 (s, C-11), 138.1 (s, SO_2_Ph-C), 135.6 (s, 4×Si-Ph-*o*-C), 135.6 (s, C-19), 134.2 (s, 2×Si-Ph-C), 133.3 (s, SO_2_Ph-*p*-C), 131.4 (s, C-15), 129.7 (s, C-7), 129.5 (s, 2×Si-Ph-*p*-C), 129.2 (s, 2×SO_2_Ph-*o*-C), 129.2 (s, 2× SO_2_Ph-*m*-C), 128.7 (s, C-6), 127.6 (s, 4×Si-Ph-*m*-C), 124.3 (s, C-18), 123.4 (s, C-14), 117.0 (s, C-10), 63.3 (s, C-9), 62.2 (s, C-1), 39.7 (s, C-2), 37.2 (s, C-4), 29.7 (s, C-12), 29.1 (s, C-8), 29.1 (s, C-16), 26.9 (s, C-3), 26.8 (s, C(CH_3_)_3_), 26.2 (s, C-5), 25.7 (s, C-13), 25.5 (s, C-17), 23.5 (s, C-25), 19.5 (s, C-24), 19.5 (s, C-23), 19.2 (s, C-22), 17.7 (s, C-21), 16.4 (s, C(CH_3_)_3_), 16.0 (s, C-20). ESI-HRMS (+) *m/z* 761.4394 (M.Na^+^), calculated 761.4377 for C_47_H_66_NaO_3_SSi^+^.

**(S,6Z,10E,14E)-3,7,11,15,19-pentamethylicosa-6,10,14,18-tetraen-1-ol (15d)**

Following exactly the same procedure as for **15c** and starting from **14d** (2.06 g, 2.79 mmol, 1.00 eq) and using TBAF (5.57 ml, 5.57 mmol, 2.00 eq, 1.00 M in THF), LiEt_3_BH (13.93 ml, 13.93 mmol, 5.00 eq, 1.00 M in THF) and (dppp)PdCl_2_ (542.4 mg, 0.92 mmol, 0.33 eq) in 40+60 ml anhydrous THF, we got **15d** (904 mg, 2.51 mmol, 90 %) as a colorless oil after purification (Rf = 0.31, DCM). ^1^H NMR (300 MHz, CDCl_3_) δ = 5.09-5.14 (m, 4H, H-6, H-10, H-14, H-18), 3.65-3.69 (m, 2H, H-1), 1.95-2.10 (m, 14H, H-5, H-8, H-9, H-12, H-13, H-16, H-17), 1.68 (s, 6H, H-24, H-25), 1.61, 1.60, 1.60 (3×s, 3×3H, H-23, H-22, H-21), 1.55-1.56 (m, 1H, H-4a), 1.30-1.41 (m, 2H, H-2), 1.15-1.24 (m, 2H, H-3, H-4b), 0.91 (d, *J* = 6.6 Hz, 3H, H-20). ^13^C NMR (75 MHz, CDCl_3_) δ = 135.2 (s, C-7), 135.1 (s, C-11), 134.9 (s, C-15), 121.2 (s, C-19), 125.4 (s, C-6), 124.4 (s, C-10), 124.2 (s, C-14), 124.2 (s, C-18), 61.2 (s, C-1), 40.0 (s, C-2), 37.7 (s, C-4), 37.5 (s, C-8), 32.0 (s, C-3), 29.3 (s, C-12), 26.8 (s, C-13), 26.6 (s, C-5), 26.6 (s, C-16), 25.7 (s, C-9), 25.3 (s, C-17), 23.4 (s, C-21), 19.5 (s, C-22), 19.5 (s, C-23), 17.7 (s, C-24), 16.0 (s, C-25), 16.0 (s, C-20). No mass could be observed by ESI-MS (+/-).

**Ammonium (S,6Z,10E,14E)-3,7,11,15,19-pentamethylicosa-6,10,14,18-tetraen-1-yl phosphate (16d)**

Following exactly the same procedure as for **16b** and starting from **15d** (900 mg, 2.50 mmol, 1.00 eq) and using CCl_3_CN (1.63 ml, 16.22 mmol, 6.50 eq) and Bu_4_NH_2_PO_4_ (1.694 mg, 4.99 mmol, 2.00 eq) in 16 ml anhydrous DCM for 8 min, we got **16d** (141 mg, 0.30 mmol, 12 %) as a colorless lyophilisat after freeze-drying (EtOAc/iPrOH/H_2_O 4:2:1, Rf = 0.48). ^1^H NMR (300 MHz, CDCl_3_) δ = 5.11-5.13 (br s, 4H, H-6, H-10, H-14, H-18), 3.87-3.92 (m, 2H, H-1), 1.95-2.08 (m, 14H, H-5, H-8, H-9, H-12, H-13, H-16, H-17), 1.67 (s, 7H, H-4a, H-24, H-25), 1.62, 1.60, 1.60 (3×s, 3×3H, H-23, H-22, H-21), 1.29-1.46 (m, 3H, H-4a, H-2), 1.14-1.21 (m, 2, H-3), 0.92 (d, *J* = 6.3 Hz, 3H, H-20). ^13^C NMR (75 MHz, CDCl_3_) δ = 136.0 (s, C-7), 135.9 (s, C-11), 135.9 (s, C-15), 132.1 (s, C-19), 126.7 (s, C-6), 125.5 (s, C-10), 125.5 (s, C-14), 125.5 (s, C-18), 64.7 (s, C-1), 40.9 (s, C-2), 39.1 (s, C-4), 39.0 (s, C-8), 38.8 (s, C-3), 32.9 (s, C-12), 30.6 (s, C-13), 27.9 (s, C-5), 27.6 (s, C-16), 27.6 (s, C-9), 26.4 (s, C-17), 25.9 (s, C-21), 23.7 (s, C-22), 19.8 (s, C-23), 17.8 (s, C-24), 16.2 (s, C-25), 16.1 (s, C-20). ^31^P NMR (122 MHz, MeOD) δ = 1.2. ESI-HRMS (-) *m/z* 439.2983 (M-[H^+^]), calculated 439.2979 for C_25_H_44_O_4_P^-^.

**(2R,3S,4R,5R,6S)-5-acetamido-6-(((2R,3S,4R,5R,6R)-5-acetamido-4-acetoxy-2-(acetoxymethyl)-6-((hydroxy((hydroxy(((S,6Z,10Z)-3,7,11,15-tetramethylhexadeca-6,10,14-trien-1-yl)oxy)phosphoryl)oxy)phosphoryl)oxy)tetrahydro-2H-pyran-3-yl)oxy)-2-(acetoxymethyl)tetrahydro-2H-pyran-3,4-diyl diacetate (17c)**

Following exactly the same procedure as for **17b** and starting from **16c** (60 mg, 0.15 mmol, 1.00 eq) and using 1,1’-carbonyldiimidazole (119.7 mg, 0.74 mmol, 5.00 eq) and **5** (316.4 mg, 0.44 mmol, 3.00 eq), we obtained **17c** (49.5 mg, 0.05 mmol, 30 %) as a colorless lyophilisat after freeze-drying (EtOAc/iPrOH/H_2_O 4:2:1, Rf = 0.33). ^1^H NMR (300 MHz, MeOD) δ = 5.51 (dd, *J* = 6.9 Hz, 3 Hz, 1H, H-1a), 5.36 (t, *J* = 9.6 Hz, 1H, H-3a), 5.24 (t, *J* = 9.3 Hz, 1H, H-3b), 5.10-5.13 (m, 3H, H-6’, H-10’, H-14’), 4.92-5.02 (m, 2H, H-1b, NHaAc), 4.82-4.84 (m, 2H, H-4b, NHbAc), 4.60 (d, *J* = 11.1 Hz, 1H, H-2a), 4.44 (dd, *J* = 12.3 Hz, 3.9 Hz, 2H, H-1’), 4.22 (dd, *J* = 10.5 Hz, 2.4 Hz, 1H, H-6a1), 4.00-4.10 (m, 4H, H-6b1, H-6a2, H-6b2, H-2b), 3.92 (t, *J* = 9.6 Hz, 1H, H-5b), 3.79-3.84 (m, 1H, H-4a), 3.62 (dd, *J* = 10.5 Hz, 8.4 Hz, 1H, H-5a), 2.11 (s, 3H, OAc), 1.98-2.06 (m, 16H, 2×3H + 10H, 2×NHAc + H-5’, H-8’, H-9’, H-12’, H-13’), 1.97 (s, 9H, 3×OAc), 1.90 (s, 3H, OAc), 1.74 (s, 1H, H-4’a), 1.68 (s, 9H, H-18’, H-19’, H-20’), 1.61 (s, 3H, H-17’), 1.34-1.48 (m, 3H, H-4’b, H-2’), 1.12-1.20 (m, 1H, H-3’), 0.92 (d, *J* = 6.6 Hz, 3H, H-16’). ^13^C NMR (75 MHz, MeOD) δ = 173.9, 173.7 (2×s, 2×NHC=OCH_3_), 172.6, 172.2, 172.2, 172.0, 171.3 (5×s, 5×OC=OCH_3_), 136.3 (s, C-7’), 135.8 (s, C-11’), 132.4 (s, C-15’), 126.7 (s, C-6’), 126.2 (s, C-10’), 125.4 (s, C-14’), 101.8 (s, C-1b), 95.8 (s, C-1a), 76.9 (s, C-4a), 73.6 (s, C-5a), 73.0 (s, C-3a), 72.8 (s, C-3b), 71.0 (s, C-5b), 70.0 (s, C-4b), 66.0 (s, C-1’), 66.0 (s, C-2’), 63.2 (s, C-6a), 63.1 (s, C-6b), 56.5 (s, C-2a), 53.4 (s, C-2b), 38.8 (s, C-4’), 33.3 (s, C-3’), 32.9 (s, C-8’), 30.6 (s, C-12’), 27.8 (s, C-5’), 27.6 (s, C-9’), 26.4 (s, C-13’), 26.0 (s, C-20’), 23.8 (s, C-19’), 23.8 (s, C-18’), 23.0, 22.8 (2×s, 2×NHC=OCH_3_), 21.2, 21.0, 20.8, 20.7, 20.6 (5×s, 5×OC=OCH_3_), 19.8 (s, C-17’), 17.9 (s, C-16’). ^31^P NMR (122 MHz, MeOD) δ = -10.4 (d, *J* = 17.8 Hz, P1), -13.2 (d, *J* = 19.3 Hz, P2). ESI-HRMS (-) *m/z* 1067.4136 (M-[H^+^]), calculated 1067.4116 for C_46_H_73_N_2_O_22_P_2_^-^

***P*1-2-acetamido-O-(2-acetamido-2-deoxy-β-D-glucopyranosyl)-(1-4)-2-deoxy-α-D-glucopyranosyl *P*2-(3S,6Z,10Z)-3,7,11,15-tetramethylhexadeca-6,10,14-trien-1-yl diphosphate (1c)**

Following exactly the same procedure as for **1b** and starting from **17c** (42.5 mg, 0.04 mmol, 1.00 eq), we obtained after 12 h reaction **1c** (34.4 mg, 0.04 mmol, quantitative yield) as a colorless lyophilisat after freeze-drying (EtOAc/iPrOH/H_2_O 4:2:1, Rf = 0.05). ^1^H NMR (300 MHz, MeOD) δ = 5.53 (dd, *J* = 6.9 Hz, 2.7 Hz, 1H, H-1a), 5.13 (br s, 3H, H-6’, H-10’, H-14’), 4.56 (d, *J* = 8.4 Hz, 1H, H-2a), 3.93-4.01 (m, 4H, H-1’, H-3a, H-3b), 3.82-3.89 (m, 3H, H-1b, H-4b, H-6a1), 3.63-3.69 (m, 3H, H-6b1, H-6a2, H-6b2), 3.48-3.59 (m, 2H, H-2b, H-5b), 3.45-3.78 (m, 2H, H-4a, H-5a), 2.05 (Br s, 11H, H-4’a, H-5’, H-8’, H-9’, H-12’, H-13’), 1.94 (s, 3H, NHC=OCH_3_), 1.68 (br s, 12H, H-17’, H-18’, H-19’, H-20’), 1.61 (s, 3H, NHC=OCH_3_), 1.34-1.47 (m, 3H, H-2’, H-4’b), 1.15-1.19 (m, 1H, H-3’), 0.93 (d, *J* = 6.3 Hz, 3H, H-16’). ^13^C NMR (75 MHz, MeOD) δ = 174.2, 173.9 (2×s, 2×NHC=OCH_3_), 136.3 (s, C-7’), 135.9 (s, C-11’), 132.4 (s, C-15’), 126.8 (s, C-6’), 126.3 (s, C-10’), 125.5 (s, C-14’), 103.1 (s, C-1b), 95.9 (s, C-1a), 81.6 (s, C-4a), 78.3 (s, C-5a), 75.9 (s, C-3a), 73.3 (s, C-3b), 72.1 (s, C-5b), 71.5 (s, C-4b), 65.7 (s, C-1’), 65.7 (s, C-2’), 62.7 (s, C-6a), 61.9 (s, C-6b), 57.6 (s, C-2a), 54.9 (s, C-2b), 39.0 (s, C-4’), 33.3 (s, C-3’), 33.0 (s, C-8’), 30.7 (s, C-12’), 27.8 (s, C-5’), 27.6 (s, C-9’), 26.5 (s, C-13’), 26.0 (s, C-20’), 23.8 (s, C-19’), 23.3 (s, C-18’), 23.1, 22.2 (2×s, 2×NHC=OCH_3_), 19.9 (s, C-17’), 17.9 (s, C-16’). ^31^P NMR (122 MHz, MeOD) δ = -10.3 (d, *J* = 19.3 Hz, P1), -12.7 (d, *J* = 19.3 Hz, P2). ESI-HRMS (-) *m/z* 857.3607 (M-[H^+^]), calculated 857.3619 for C_36_H_63_N_2_O_17_P_2_^-^.

**(2R,3S,4R,5R,6S)-5-acetamido-6-(((2R,3S,4R,5R,6R)-5-acetamido-4-acetoxy-2-(acetoxymethyl)-6-((hydroxy((hydroxy(((S,6Z,10E,14E)-3,7,11,15,19-pentamethylicosa-6,10,14,18-tetraen-1-yl)oxy)phosphoryl)oxy)phosphoryl)oxy)tetrahydro-2H-pyran-3-yl)oxy)-2-(acetoxymethyl)tetrahydro-2H-pyran-3,4-diyl diacetate (17d)**

Following exactly the same procedure as for **17b** and starting from **16d** (68 mg, 0.14 mmol, 1.00 eq) and using 1,1’-carbonyldiimidazole (116.2 mg, 0.72 mmol, 5.00 eq) and **5** (307.1 mg, 0.43 mmol, 3.00 eq), we obtained **17d** (84.5 mg, 0.07 mmol, 50 %) as a colorless lyophilisat after freeze-drying (EtOAc/iPrOH/H_2_O 4:2:1, Rf = 0.37). ^1^H NMR (300 MHz, MeOD) δ = 5.54 (dd, *J* = 7.2 Hz, 3 Hz, 1H, H-1a), 5.34 (t, *J* = 9.9 Hz, 1H, H-3a), 5.10-5.15 (m, 5H, H-3b, H-6’, H-10’, H-14’, H-18’), 4.96 (t, *J* = 9.6 Hz, 1H, H-1b), 4.79 (d, *J* = 8.1 Hz, 1H, H-4b), 4.53 (d, *J* = 11.4 Hz, 1H, H-2a), 4.44 (dd, *J* = 12.3 Hz, 3.9 Hz, 1H,H-6a1), 4.21 (tt, *J* = 10.5 Hz, 2.7 Hz, 1H, H-6b1), 4.13 (d, *J* = 10.2 Hz, 1H, H-6a2), 3.99-4.06 (m, 4H, H-1’, H-6b2, H-2b), 3.88 (t, *J* = 9.3 Hz, 1H, H-5b), 3.79 (tt, *J* = 10.5 Hz, 3.0 Hz, 1H, H-4a), 3.62 (dd, *J* = 10.2 Hz, 8.7 Hz, 1H, H-5a), 2.02-2.08 (br m, 18H, H-5’, H-8’, H-9’, H-12’, H-13’, H-16’, H-17’, OAc, H-4’), 1.94-1.98 (br m, 15H, 4×OAc + NHAc), 1.90 (s, 3H, NHAc), 1.66, 1.61, 1.59 (3×s, 6H + 3H + 6H, H-21’, H-22’, H-23’, H-24’, H-25’), 1.28-1.45 (m, 3H, H-4’b, H-2’), 1.10-1.20 (m, 1H, H-3’), 0.89 (d, *J* = 6.3 Hz, 3H, H-20’). ^13^C NMR (75 MHz, MeOD) δ = 173.9, 173.6 (2×s, 2×NHC=OCH_3_), 172.5, 172.2, 172.0, 171.8, 171.3 (5×s, 5×OC=OCH_3_), 136.1 (s, C-7’), 136.0 (s, C-11’), 135.9 (s, C-15’), 132.1 (s, C-19’), 126.7 (s, C-6’), 125.5 (s, C-10’), 125.5 (s, C-14’), 121.7 (s, C-18’), 101.8 (s, C-1b), 95.8 (s, C-1a), 77.0 (s, C-4a), 73.7 (s, C-5a), 73.0 (s, C-3a), 72.8 (s, C-3b), 70.9 (s, C-5b), 70.0 (s, C-4b), 65.8 (s, C-1’), 65.8 (s, C-2’), 63.3 (s, C-6a), 63.1 (s, C-6b), 56.5 (s, C-2a), 53.4 (s, C-2b), 40.9 (s, C-4’), 38.9 (s, C-3’), 38.8 (s, C-8’), 33.7 (s, C-12’), 33.0 (s, C-16’), 27.9 (s, C-5’), 27.6 (s, C-9’), 27.6 (s, C-13’), 26.4 (s, C-17’), 26.0 (s, C-25’), 23.8 (s, C-24’), 23.1, 22.9 (2×s, 2×NHC=OCH_3_), 21.2, 21.0, 20.8, 20.7, 20.6 (5×s, 5×OC=OCH_3_), 19.8 (s, C-23’), 16.3 (s, C-22’), 16.2 (s, C-21’). ^31^P NMR (122 MHz, MeOD) δ = -10.0 (d, *J* = 19.3 Hz, P1), -13.1 (d, *J* = 19.3 Hz, P2). ESI-HRMS (-) *m/z* 1135.4762 (M-[H^+^]), calculated 1135.4772 for C_51_H_81_N_2_O_22_P_2_^-^.

***P*1-2-acetamido-O-(2-acetamido-2-deoxy-β-D-glucopyranosyl)-(1-4)-2-deoxy-α-D-glucopyranosyl *P*2-(S,6Z,10E,14E)-3,7,11,15,19-pentamethylicosa-6,10,14,18-tetraen-1-yl diphosphate (1d)**

Following exactly the same procedure as for **1b** and starting from **17d** (74.5 mg, 0.06 mmol, 1.00 eq), we obtained after 12 h reaction **1d** (61 mg, 0.06 mmol, quantitative yield) as a colorless lyophilisat after freeze-drying (EtOAc/iPrOH/H_2_O 4:2:1, Rf = 0.05). ^1^H NMR (300 MHz, MeOD) δ = 5.56 (dd, *J* = 7.2 Hz, 3.0 Hz, 1H, H-1a), 5.08-5.13 (br m, 4H, H-6’, H-10’, H-14’, H-18’), 4.54 (d, *J* = 8.4 Hz, 1H, H-2a), 4.00-4.04 (m, 3H, H-1’, H-3a), 3.85-3.92 (m, 2H, H-3b, H-1b), 3.78-3.80 (m, 1H, H-4b), 3.30 (d, *J* = 10.2 Hz, 2H, H-6a1, H-6b1), 3.65-3.68 (m, 2H, H-6a2, H-6b2), 3.53-3.63 (m, 2H, H-2b, H-5b), 3.47-3.50 (m, 1H, H-4a), 3.35-3.36 (m, 1H, H-5a), 2.02-2.10 (Br m, 15H, H-4’a, H-5’, H-8’, H-9’, H-12’, H-13’, H-16’, H-17’), 1.98, 1.94 (2×s, 2×3H, 2×NHC=OCH_3_), 1.66, 1.61, 1.59 (3×s, 6H + 3H + 6H, H-21’, H-22’, H-23’, H-24’, H-25’), 1.29-1.45 (m, 3H, H-2’, H-4’b), 1.10-1.17 (m, 1H, H-3’), 0.90 (d, *J* = 6.3 Hz, 3H, H-20’). ^13^C NMR (75 MHz, MeOD) δ = 174.1, 173.8 (2×s, 2×NHC=OCH_3_), 136.1 (s, C-7’), 135.9 (s, C-11’), 135.9 (s, C-15’), 132.1 (s, C-19’), 126.6 (s, C-6’), 125.5 (s, C-10’), 125.5 (s, C-14’), 121.7 (s, C-18’), 103.1 (s, C-1b), 96.0 (s, C-1a), 81.5 (s, C-4a), 78.2 (s, C-5a), 75.9 (s, C-3a), 73.3 (s, C-3b), 72.0 (s, C-5b), 71.3 (s, C-4b), 65.7 (s, C-1’), 65.7 (s, C-2’), 62.6 (s, C-6a), 61.7 (s, C-6b), 57.5 (s, C-2a), 54.8 (s, C-2b), 40.9 (s, C-4’), 40.0 (s, C-3’), 38.8 (s, C-8’), 33.0 (s, C-12’), 30.6 (s, C-16’), 27.9 (s, C-5’), 27.6 (s, C-9’), 27.6 (s, C-13’), 26.4 (s, C-17’), 26.0 (s, C-25’), 23.8 (s, C-24’), 23.3, 23.1 (2×s, 2×NHC=OCH_3_), 19.8 (s, C-23’), 17.9 (s, C-22’), 16.2 (s, C-21’), 16.2 (s, C-20’). ^31^P NMR (122 MHz, MeOD) δ = -10.2 (d, *J* = 17.9 Hz, P1), -12.6 (d, *J* = 17.7 Hz, P2). ESI-HRMS (-) *m/z* 925.4233 (M-[H^+^]), calculated 925.4227 for C_41_H_71_N_2_O_17_P_2_^-^.

***Synthesis and characterization of LLO inhibitors***

**(2R,3S,4R,5R,6R)-5-acetamido-2-(acetoxymethyl)-6-chlorotetrahydro-2H-pyran-3,4-diyl diacetate (19)**

**18** (2.50 g, 11.30 mmol, 1.00 eq) was suspended in acetyl chloride (4.82 ml, 67.81 mmol, 6.00 eq) at 4°C and 5 ml DCM was added. The reaction mixture was stirred at RT for 5 days under Ar. The resulting mixture was dissolved in 150 ml CHCl_3_, washed with 3×150 ml ice water, 2×150 ml saturated NaHCO_3_ and 150 ml brine, dried over Na_2_SO_4_ and concentrated to yield **19** (3.87 g, 10.58 mmol, 94 %) as a colorless foam which was used in the next step without further purifications (Rf = 0.31, DCM/MeOH 98:2). ^1^H NMR (300 MHz, CDCl_3_) δ = 6.17 (d, *J* = 3.6 Hz, 1H, H-1), 5.88 (d, *J* = 8.7 Hz, 1H, NH), 5.31 (t, *J* = 9.6 Hz, 1H, H-3), 5.19 (t, *J* = 9.6 Hz, 1H, H-4), 4.52 (ddd, *J* = 12.6 Hz, 9 Hz, 3.9 Hz, 1H, H-2), 4.24-4.28 (m, 2H, H-5, H-6a), 4.11 (d, *J* = 10.2 Hz, 1H, H-6b), 2.08, 2.03, 2.03 (3×s, 3×3H, 3×OAc), 1.97 (s, 3H, NHAc). ^13^C NMR (75 MHz, CDCl_3_) δ = 171.4, 170.5, 170.1 (3×s, 3×OC=O), 169.1 (s, NHC=O), 93.7 (s, C-1), 70.9 (s, C-5), 70.2 (s, C-3), 67.0 (s, C-4), 6.2 (s, C-6), 53.5 (s, C-2), 23.0 (s, NHC=OCH_3_), 2×20.7, 20.5 (3×s, 3×OC=OCH_3_). ESI-MS (+) *m/z* calculated 388.08 (M.Na^+^), found 388.08 for C_14_H_20_ClNNaO_8_^+^.

**(2R,3S,4R,5S,6R)-5-acetamido-2-(acetoxymethyl)-6-allyltetrahydro-2H-pyran-3,4-diyl diacetate (20)**

A suspension **19** (6.00 g, 16.43 mmol, 1.00 eq), allyltributyltin (25.47 ml, 82.17 mmol, 5.00 eq) and AIBN (405 mg, 2.47 mmol, 0.15 eq) in 33 ml dry toluene was stirred overnight at 85°C under an Ar atmosphere. The mixture was then concentrated *in vacuo,* purified by flash column chromatography on silica gel (Rf = 0.44, EtOAc) and crystallized from EtOAc/Hexane 1:3 to yield **20** (6.09 g, 16.40 mmol, 58 %) as a colorless solid. ^1^H NMR (300 MHz, CDCl_3_) δ = 5.89 (d, *J* = 8.1 Hz, 1H, NH), 5.68-5.82 (m, 1H, H-8), 5.07-5.15 (m, 2H, H-9), 5.03 (d, *J* = 7.5 Hz, 1H, H-3), 4.94 (t, *J* = 6.9 Hz, 1H, H-4), 4.19-4.33 (m, 3H, H-6a, H-2, H-1), 4.10 (dd, *J* = 12.0 Hz, 3.6 Hz, 1H, H-6b), 3.85-3.91 (m, 1H, H-5), 2.35-2.44 (m, 1H, H-7a), 2.28-2.30 (m, 1H, H-7b), 2.07, 2.07, 2.06 (3xs, 3x3H, 3xOAc), 1.96 (s, 3H, NHAc). ^13^C NMR (75 MHz, CDCl_3_) δ = 171.0, 170.6, 169.6, 169.0 (4xs, NHC=OCH_3_ + 3xOC=OCH_3_), 133.3 (s, C-8), 117.7 (s, C-9), 70.9 (s, C-5), 70.7 (s, C-1), 70.1 (s, C-3), 67.9 (s, C-4), 61.6 (s, C-6), 50.6 (s, C-2), 32.2 (s, C-7), 23.2 (s, NHC=OCH_3_), 20.8, 20.7, 20.7 (3xs, 3xOC=OCH_3_). ESI-HRMS (+) *m/z* calculated 372.1653 (M+[H^+^]), found 372.1642 for C_17_H_26_NO_8_^+^.

**N-((2R,4aR,6R,7R,8R,8aS)-6-allyl-8-hydroxy-2-phenylhexahydropyrano[3,2-d][1,3]dioxin-7-yl)acetamide (21)**

**20** (11.30 g, 30.43 mmol, 1.00 eq) was dissolved in 60 ml MeOH and sodium methoxide (6.96 ml, 30.43 mmol, 1.00 eq, 25 wt % in MeOH) was added. The reaction mixture was stirred at RT under an Ar atmosphere overnight. The reaction mixture was neutralized using Amberlyst IR-15 ion exchange resin and concentrated to dryness. The residue was dissolved under an Ar atmosphere in benzaldehyde (99 ml, 0.97 mol, 32.00 eq). At 0°C, TFA (5.65 ml, 76.07 mmol, 2.50 eq) was added dropwise and the reaction mixture was stirred while warming up to RT over 3 h, concentrated to dryness, triturated several times with hexane and then water and concentrated again to dryness to yield **21** (10.10 g, 30.30 mmol, quantitative yield) as a colorless solid which was used in the next step without further purification (Rf = 0.19, DCM/MeOH 96:4). ^1^H NMR (400 MHz, MeOD) δ = 7.50-7.52 (m, 2H, Ar-H), 7.34-7.35 (m, 3H, Ar-H), 5.75-5.84 (m, 1H, H-8), 5.60 (s, 1H, H-10), 5.15 (dd, *J* = 17.2 Hz, 2.0 Hz, 1H, H-9a), 5.07 (dd, *J* = 9.2 Hz, 1.2 Hz, 1H, H-9b), 4.24-4.30 (m, 1H, H-6a), 4.10-4.17 (m, 2H, H-1, H-2), 3.87 (dd, *J* = 10.4 Hz, 9.2 Hz, 1H, H-3), 3.71 (t, *J* = 10.0 Hz, 1H, H-6b), 3.61 (dt, *J* = 9.6 Hz, 4.8 Hz, 1H, H-5), 3.52 (t, *J* = 8.8 Hz, 1H, H-4), 2.56-2.61 (m, 1H, H-7a), 2.28-2.33 (m, 1H, H-7b), 1.98 (s, 3H, NHAc). ^13^C NMR (101 MHz, MeOD) δ = 172.2 (s, NHC=OCH_3_), 137.5 (s, Ar-C), 134.5 (s, C-8), 128.5, 2x127.6, 2x126.1 (5xs, 5xAr-C), 115.8 (s, C-9), 101.7 (s, C-10), 83.1 (s, C-4), 74.3 (s, C-1), 68.8 (s, C-6), 67.9 (s, C-3), 63.6 (s, C-5), 54.3 (s, C-2), 30.4 (s, C-7), 21.2 (s, NHC=OCH_3_).

**N-((2R,4aR,6R,7S,8R,8aS)-6-allyl-8-(benzyloxy)-2-phenylhexahydropyrano[3,2-d][1,3]dioxin-7-yl)acetamide (22)**

**21** (8.70 g, 26.10 mmol, 1.00 eq) was  dissolved in 265 ml DMF and NaH (2.00 g, 52.19 mmol, 2.00 eq) was added at RT under an Ar atmosphere. The reaction mixture was stiired at RT for 30 min, cooled down to 0°C and benzyl bromide (6.21 ml, 52.19 mmol, 2.00 eq) was added dropwise. The reaction mixture was stirred for 4 h at RT, quenched by addition of 10 ml MeOH and stirred for 1 h. The mixture was partially concentrated *in vacuo*, diluted with 550 ml EtOAc and extracted with 550 ml water and brine, dried over Na_2_SO_4_, filtered and concentrated under reduced pressure. The crude residue was purified by flash column chromatography on silica gel (DCM/EtOAc 95:5 to 90:10 to 80:20, Rf = 22, DCM/EtOAc 90:10) to yield **22** (4.98 g, 11.76 mmol, 45 %) as a colorless solid. ^1^H NMR (300 MHz, CDCl_3_) δ = 7.50-7.53 (m, 2H, Ar-H), 7.36-7.42 (m, 8H, Ar-H), 5.64-5.76 (m, 1H, H-8), 5.60 (s, 1H, H-10), 5.02-5.10 (m, 3H, NH, H-9a, H-9b), 4.95 (d, *J* = 12.0 Hz, 1H, H-11a), 4.68 (d, *J* = 12.0 Hz, 1H, H-11b), 4.39-4.58 (m, 1H, H-1), 4.24 (dd, *J* = 10.2 Hz, 4.5 Hz, 1H, H-6a), 4.09-4.16 (m, 1H, H-2), 3.80-3.83 (m, 1H, H-4), 3.68-3.77 (m, 2H, H-3, H-6b), 3.62-3.65 (m, 1H, H-5), 2.21-2.37 (m, 1H, H-7a), 2.14-2.20 (m, 1H, H-7b), 1.84 (s, 3H, NHAc). ^13^C NMR (75 MHz, CDCl_3_) δ = 170.1 (s, NHC=OCH_3_), 138.3, 137.4 (2xs, 2xAr-C), 133.8 (s, C-8), 129.0, 128.7, 128.5, 128.3, 128.2, 126.0 (6xs, 6xAr-C), 117.3 (s, C-9), 101.3 (s, C-10), 83.3 (s, C-4), 74.9 (s, C-3), 73.5 (2xs, C-1, C-11), 69.4 (s, C-6), 63.9 (s, C-5), 53.2 (s, C-2), 31.2 (s, C-7), 23.2 (s, NHC=OCH_3_).

**N-((2R,4aR,6R,7S,8R,8aS)-8-(benzyloxy)-2-phenyl-6-((E)-prop-1-en-1-yl)hexahydropyrano[3,2-d][1,3]dioxin-7-yl)acetamide (23)**

(1,5-Cyclooctadiene)bis(methyldiphenylphosphine)iridium(I) hexafluorophosphate (995 mg, 1.18 mmol, 0.10 eq) was dissolved in 140 ml THF and activated under an H_2_ atmosphere until the opaque red suspension became a clear, slightly orange solution. Ar was then bubbled through the solution for 5 minutes to remove any H_2_. The activated catalyst was then added to a solution of **22** (4.98 g, 11.76 mmol, 1.00 eq) in 800 ml THF and the reaction mixture was stirred at RT under an Ar atmosphere for 2 days. The orange mixture was concentrated under reduced pressure and purified by flash column chromatography on silica gel (DCM/EtOAc 9:1 to 7:3, Rf = 0.26, DCM/EtOAc 9:1) to yield **23** (4.97 g, 11.74 mmol, quantitative yield) as a yellow solid. ^1^H NMR (400 MHz, CDCl_3_) δ = 7.50-7.52 (m, 2H, Ar-H), 7.35-7.39 (m, 8H, Ar-H), 5.78-5.87 (m, 1H, H-8), 5.60 (s, 1H, H-10), 5.56-5.62 (m, 1H, H-7), 4.96-4.98 (m, 1H, NH), 4.95 (d, *J* = 12.4 Hz, 1H, H-11a), 4.66 (d, *J* = 13.2 Hz, 1H, H-11b), 4.64-4.68 (m, 1H, H-1), 4.23-4.28 (m, 1H, H-6a), 4.17-4.22 (m, 1H, H-2), 3.79-3.83 (m, H-4), 3.73-3.82 (m, 2H, H-5, H-6b), 3.62 (dd, *J* = 10.0 Hz, 8.8 Hz, 1H, H-3), 1.85 (s, 3H, NHAc), 1.74 (d, *J* = 6.4 Hz, 1H, H-9). ^13^C NMR (101 MHz, CDCl_3_) δ = 169.9 (s, NHC=OCH_3_), 138.7, 137.7 (2xs, 2xAr-C), 134.2 (s, C-8), 129.1, 128.7, 128.4, 128.3, 128.1, 126.2 (6xs, 6xAr-C), 123.9 (s, C-7), 101.5 (s, C-10), 83.7 (s, C-4), 76.0 (s, C-3), 75.8 (s, C-1), 73.8 (s, C-11), 69.6 (s, C-6), 65.1 (s, C-5), 52.9 (s, C-2), 23.4 (s, NHC=OCH_3_), 18.4 (s, C-9). ESI-MS (+) *m/z* calculated 424.21 (M+[H^+^]), found 424.21 for C_25_H_30_NO_5_^+^.

**N-((2R,3S,4R,5S,6R)-4-(benzyloxy)-6-((benzyloxy)methyl)-5-hydroxy-2-((E)-prop-1-en-1-yl)tetrahydro-2H-pyran-3-yl)acetamide (24)**

To a solution of **23** (4.98 g, 11.76 mmol, 1.00 eq), 4Å crushed molecular sieves  in 100 ml dry DCM was added at 0°C under an Ar atmosphere Et_3_SiH (9.34 ml, 58.80 mmol, 5.00 eq). Right after, BF_3_.OEt_2_ (7.26 ml, 58.80 mmol, 5.00 eq) was added dropwise and the reaction mixture was allowed to stir at RT for 4 h, was then diluted with DCM, filtered on celite, washed two times with NaHCO_3_, once with brine, dried over Na_2_SO_4_, filtered and concentrated. The residue was purified by flash column chromatography on silica gel (DCM/EtOAc 8:2 to 7:3 to 6:4, Rf = 0.20, DCM/EtOAc 7:3) to yield **24** (2.80 g, 11.75 mmol, 56 %) as a colorless sticky solid. ^1^H NMR (400 MHz, CDCl_3_) δ = 7.28-7.38 (m, 10H, Ar-H), 5.74-5.83 (m, 1H, H-8), 5.47-5.56 (m, 2H, H-7, NH), 4.76 (d, *J* = 12.0 Hz, 1H, H-11a), 4.69 (d, *J* = 12.0 Hz, 1H, H-11b), 4.58 (d, *J* = 12.0 Hz, 1H, H-10a), 4.54 (d, *J* = 12.0 Hz, 1H, H-10b), 4.50-4.53 (m, 1H, H-1), 4.11-4.19 (m, 1H, H-2), 3.82-3.88 (m, 2H, H-4, H-5), 3.76-3.80 (m, 1H, H-6a), 3.67-3.72 (m, 1H, H-6b), 3.51 (dd, *J* = 8.0 Hz, 6.8 Hz, 1H, H-3), 1.86 (s, 3H, NHAc), 1.72 (d, *J* = 6.4 Hz, 1H, H-9). ^13^C NMR (101 MHz, CDCl_3_) δ = 169.8 (s, NHC=OCH_3_), 138.4, 137.8 (2xs, 2xAr-C), 132.3 (s, C-8), 128.6, 128.4, 127.9, 127.9, 127.8, 127.8 (6xs, 6xAr-C), 124.8 (s, C-7), 78.4 (s, C-3), 73.7 (s, C-10), 73.5 (s, C-5), 72.9 (s, C-11), 72.8 (s, C-1), 71.8 (s, C-4), 705 (s, C-6), 50.6 (s, C-2), 23.3 (s, NHC=OCH_3_), 18.1 (s, C-9). ESI-MS (+) *m/z* calculated 426.23 (M+[H^+^]), found 426.23 for C_25_H_32_NO_5_^+^.

**(3R,4R,5S,6R)-6-(acetoxymethyl)-3-(((2,2,2-trichloroethoxy)carbonyl)amino)tetrahydro-2H-pyran-2,4,5-triyl triacetate (26)**

Troc-Cl (9.00 ml, 66.78 mmol, 1.20 eq) was added dropwise ar RT to a solution of **25** (12 g, 55.65 mmol, 1.00 eq) and sodium carbonate (14 g, 166.95 mmol, 3.00 eq) in 110 ml water. The reaction mixture was stirred for 1 h and neutralized with 1 M HCl and then freeze-dried.
Pyridine (56 ml, 695.63 mmol, 12.50 eq) and acetic anhydride (31.5 ml, 333.90 mmol, 6 eq) were added to the residue at 0°C under an Ar atmosphere and the reaction mixture was stirred at RT overnight and concentrated *in vacuo*. Then 100 ml toluene was added and the mixture was again concentrated *in vacuo*. The residue was purified by flash column chromatography on silica gel (EtOAc/hexane 4:6 to 5:5, Rf = 0.21, EtOAc/hexane 2:8) to yield **26** (26.72 g, 32.48 mmol, 92 %) as a colorless foam. ^1^H NMR (300 MHz, CDCl_3_) δ = 6.23 (d, *J* = 3.6 Hz, 1H, H-1), 5.14-5.31 (m, 3H, H-3, H-4, NH), 4.81 (d, *J* = 12.0 Hz, 1H, H-7a), 4.61 (d, *J* = 12.0 Hz, 1H, H-7b), 4.16-4.30 (m, 2H, H-2, H-6a), 3.98-4.08 (m, 2H, H-5, H-6b), 2.20, 2.08, 2.04, 2.03 (4xs, 4xH, 4xOAc). ^13^C NMR (75 MHz, CDCl_3_) δ = 171.3, 170.7, 169.2, 168.6 (4xs, 4xOC=OCH_3_), 154.0 (s, NHC=OOCH_2_CCl_3_), 117.5 (s, CH_2_CCl_3_), 90.4 (s, C-1), 74.7 (s, CH_2_CCl_3_), 70.4 (s, C-3), 69.8 (s, C-5), 67.5 (s, C-4), 61.5 (s, C-6), 53.2 (s, C-2), 20.9, 20.7, 20.7, 20.6 (4xs, 4xOC=OCH_3_).

**(2R,3S,4R,5R)-2-(acetoxymethyl)-6-hydroxy-5-(((2,2,2-trichloroethoxy)carbonyl)amino)tetrahydro-2H-pyran-3,4-diyl diacetate (27)**

**26** (26.56 g, 50.81 mmol, 1.00 eq) was dissolved in 260 ml dry DMF and hydrazine acetate (6.08 g, 66.06 mmol, 1.30 eq) was added. The reaction mixture was then stirred at RT for 2h under an Ar atmosphere and was then diluted in 1 l EtOAc, washed with 2x400 ml brine and 2x400 ml water, dried over Na_2_SO_4_ and concentrated *in* vacuo to yield **27** (23.63 g, 49.16 mmol, 97 %) as a colorless solid which was used in the next step without further purification (Rf = 0.36, EtOAc/hexane 4:6). ^1^H NMR (300 MHz, CDCl_3_) δ = 5.52 (d, *J* = 3.6 Hz, 1H, H-1), 5.28-5.31 (m, 2H, H-3, NH), 5.07-5.14 (m,1H, H-4), 4.49 (d, *J* = 12.0 Hz, 1H, H-7a), 4.62 (d, *J* = 12.0 Hz, 1H, H-7b), 4.20-4.24 (m, 2H, H-5, H-6a), 4.12-4.15 (m, 2H, H-2, H-6b), 2.08, 2.02, 1.99 (3xs, 3x3H, 3xOAc). ^13^C NMR (75 MHz, CDCl_3_) δ = 171.1, 171.0, 169.5 (3xs, 3xOC=OCH_3_), 154.3 (s, NHC=OOCH_2_CCl_3_), 95.4 (s, CH_2_CCl_3_), 91.7 (s, C-1), 74.6 (s, CH_2_CCl_3_), 70.8 (s, C-3), 68.4 (s, C-4), 67.6 (s, C-5), 62.1 (s, C-6), 54.2 (s, C-2), 20.8, 20.7, 20.7 (3xs, 3xOC=OCH_3_). ESI-MS (-) *m/z* calculated 478.01 (M-[H^+^]), found 478.01 for C_15_H_19_Cl_3_NO_10_^-^.

**(2R,3S,4R,5R,6R)-2-(acetoxymethyl)-6-(2,2,2-trichloro-1-iminoethoxy)-5-(((2,2,2-trichloroethoxy)carbonyl)amino)tetrahydro-2H-pyran-3,4-diyl diacetate (28)**

**27** (23.57 g, 49.04 mmol, 1.00 eq) was dissolved in 140 ml DCM and trichloroacetonitrile (49.2 ml, 490.36 mmol, 10.00 eq) and DBU (1.83 ml, 12.26 mmol, 0.25 eq) were added at RT under an Ar atmosphere. The reaction mixture was stirred at this temperature for 20 min and was then concentrated under reduced pressure. The residue was purified by flash column chromatography on silica gel (EtOAc/hexane 1:2 + 0.1 % Et_3_N, Rf = 0.23, EtOAc/hexane 1:9) to yield **28** (16.05 g, 25.68 mmol, 52 %) as a yellow solid. ^1^H NMR (300 MHz, CDCl_3_) δ = 8.80 (s, 1H, C=NH), 6.41 (d, *J* = 3.6 Hz, 1H, H-1), 5.33 (dd, *J* = 20.1 Hz, 9.6 Hz, 1H, H-3), 5.19-5.23 (m, 2H, H-4, NH), 4.71 (d, *J* = 1.5 Hz, 2H, H-7), 4.24-4.31 (m, 2H, H-2, H-6a), 4.11-4.16 (m, 2H, H-5, H-6b), 2.07, 2.05, 2.05 (3xs, 3x3H, 3xOAc). ^13^C NMR (75 MHz, CDCl_3_) δ = 171.1, 170.6, 169.3 (3xs, 3xOC=OCH_3_), 160.4 (s, OC=NCCl_3_), 154.1 (s, NHC=OOCH_2_CCl_3_), 95.2 (s, CH_2_CCl_3_), 94.5 (s, OC=NCCl_3_), 90.7 (s, C-1), 74.7 (s, CH_2_CCl_3_), 2x70.3 (2xs, C-3, C-4), 67.4 (s, C-5), 61.4 (s, C-6), 53.9 (s, C-2), 20.7, 20.7, 20.6 (3xs, 3xOC=OCH_3_).

**(2R,3S,4R,5R,6S)-6-(((2R,3S,4R,5S,6R)-5-acetamido-4-(benzyloxy)-2-((benzyloxy)methyl)-6-((E)-prop-1-en-1-yl)tetrahydro-2H-pyran-3-yl)oxy)-2-(acetoxymethyl)-5-(((2,2,2-trichloroethoxy)carbonyl)amino)tetrahydro-2H-pyran-3,4-diyl diacetate (29)**

TMSOTf (0.59 ml, 3.24 mmol, 0.50 eq) in 5.4 ml dry DCM was added dropwise at 0°C to a solution of **24** (2.76 g, 6.49 mmol, 1.00 eq) and **28** (8.11 g, 12.97 mmol, 2.00 eq) in 130 ml dry DCM under an Ar atmosphere. The reaction mixture was then stirred at RT for 18 h. 2.6 ml N,N-ethyldiisopropylamine was added dropwise to the reaction mixture which was then filtered on celite and concentrated to dryness. The residue was purified by flash column chromatography on silica gel (EtOAc/hexane 3:7 to 5:5 to 7:3, Rf = 0.50, EtOAc/hexane 4:6) to yield **29** (5.24 g, 5.90 mmol, 91 %) as a yellow powder. ^1^H NMR (400 MHz, CDCl_3_) δ = 7.27-7.37 (m, 10H, Ar-H), 6.59 (d, *J* = 10.0 Hz, 1H, NH), 5.69 (ddd, *J* = 15.2 Hz, 6.4 Hz, 1.2 Hz, 1H, H-8a), 5.35 (dd, *J* = 5.6 Hz, 1.6 Hz, 1H, H-7a), 4.97-5.10 (m, 4H), 4.78 (d, *J* = 11.6 Hz, 1H), 4.62-4.68 (m, 2H), 4.55-4.59 (m, 2H), 4.46 (d, *J* = 12.0 Hz, 1H), 4.33 (d, *J* = 5.2 Hz, 1H, H-1), 4.22-4.29 (m, 3H), 4.05 (dd, *J* = 12.0 Hz, 2.4 Hz, 1H), 3.89-3.91 (m, 1H), 3.71-3.81 (m, 4H), 3.55-3.58 (m, 1H), 2.05, 2.05, 2.04, 2.04 (4xs, 4x3H, 3xOAc + NHAc), 1.67 (d, *J* = 6.0 Hz, 3H, H-9a). ^13^C NMR (75 MHz, CDCl_3_) δ = 171.1, 170.8, 170.6 (3xs, 3xOC=OCH_3_), 169.4 (s, NHC=OCH_3_), 155.0 (s, NHC=OOCH_2_CCl_3_), 138.1, 137.9 (2xs, 2xAr-C), 129.2 (s, C-8a), 128.6, 128.5, 128.0, 127.9, 127.8, 127.5, 127.4 (s, C-7a), 101.1, 95.2, 75.6, 75.2, 74.8 (s, CH_2_), 73.5 (s, CH_2_), 73.0, 72.2 (s, CH_2_), 71.8, 71.7, 68.6 (s, C-1a), 68.3, 67.7 (s, CH_2_), 61.9 (s, CH_2_), 56.1, 47.7, 22.9 (s, NHC=OCH_3_), 20.7, 20.6, 20.6 (3xs, 3xOC=OCH_3_), 18.0 (s, C-9a). ESI-MS (+) *m/z* calculated 887.23 (M+[H^+^]), found 887.23 for C_40_H_50_Cl_3_N_2_O_14_^+^.

**(2R,3S,4R,5R,6S)-5-acetamido-6-(((2R,3S,4R,5S,6R)-5-acetamido-4-(benzyloxy)-2-((benzyloxy)methyl)-6-((E)-prop-1-en-1-yl)tetrahydro-2H-pyran-3-yl)oxy)-2-(acetoxymethyl)tetrahydro-2H-pyran-3,4-diyl diacetate (30)**

**29** (4.18 g, 4.71 mmol, 1.00 eq) was dissolved in 84 ml dry DCM and 176 ml Ac2O was added at RT under an Ar atmosphere. Then, Zn dust (23.12 g, 353.56 mmol, 75.00 eq) was added and the reaction mixture was stirred at RT overnight, filtered on celite and concentrated to dryness. The residue was purified by flash column chromatography on silica gel (EtOAc/hexane 8:2, Rf = 0.23, EtOAc/hexane 7:3) to yield **30** (2.35 g, 3.11 mmol, 66 %) as a colorless solid. ^1^H NMR (400 MHz, CDCl_3_) δ = 7.30-7.36 (m, 10H, Ar-H), 5.67 (dt, *J* = 14.0 Hz, 8.0 Hz, 1H, H-8a), 5.53 (d, *J* = 8.8 Hz, 1H, NH), 5.36 (dd, *J* = 15.6 Hz, 6.0 Hz, 1H, H-7a), 5.10 (t, *J* = 10.0 Hz, 1H), 4.96 (t, *J* = 10.0 Hz, 1H), 4.69 (d, *J* = 12.0 Hz, 1H), 4.47-4.57 (m, 3H), 4.23-4.30 (m, 3H, H-1a), 4.05-4.18 (m, 3H), 3.72-3.90 (m, 5H), 3.56 (d, *J* = 9.6 Hz, 1H), 2.20 (s, 3H, NHAc), 2.07, 2.05, 2.03 (3xs, 3x3H, 3xOAc), 1.96 (s, 3H, NHAc), 1.67 (d, *J* = 6.0 Hz, 3H, H-9a). ^13^C NMR (101 MHz, CDCl_3_) δ = 170.6, 170.4, 170.4, 169.7, 168.2 (5xs, 3xOC=OCH_3_+ 2xNHC=OCH_3_), 137.1, 136.8 (2xs, 2xAr-C), 128.2 (s, C-8a), 127.5, 127.4, 126.9, 126.8, 126.5, 126.3 (s, C-7a), 99.8, 74.4, 74.1, 72.6, 71.4, 71.3, 71.1, 71.0, 67.5 (s, C-1a), 67.5, 67.0, 66.4, 60.9, 52.9, 46.9, 22.6, 21.8 (2xs, 2xNHC=OCH_3_), 19.8, 19.7, 19.6 (3xs, 3xOC=OCH_3_), 17.0 (s, C-9a). ESI-HRMS (+) *m/z* calculated 777.3205 (M+[Na^+^]), found 777.3206 for C_39_H_50_N_2_NaO_13_^+^.

**(2R,3S,4R,5R,6S)-5-acetamido-6-(((2R,3S,4R,5R,6S)-5-acetamido-4-(benzyloxy)-2-((benzyloxy)methyl)-6-((diethoxyphosphoryl)(hydroxy)methyl)tetrahydro-2H-pyran-3-yl)oxy)-2-(acetoxymethyl)tetrahydro-2H-pyran-3,4-diyl diacetate (31)**

A steady stream of ozone in oxygen generated with an ozonizer was bubbled through a solution of **30** (1.34 g, 1.77 mmol, 1.00 eq) in 24 ml DCM/MeOH 20:4 for 30 min at -78°C. Then, the color of the solution turned blue, meaning an excess of ozone and a total conversion of the reaction. The reaction mixture was stirred under an Ar atmosphere at -78°C for 10 min. A steady sream of oxygen was bubbled through the reaction mixture for 10 min to remove the excess of ozone in the solution. Under an Ar atmosphere, dimethyl sulfide (0.66 ml, 8.86 mmol, 5.00 eq) was added to the reaction mixture which was allowed to warm up to RT overnight and was then concentrated to dryness under reduced pressure. The residue (Rf = 0.13, DCM/MeOH 96:4) was used in the next step without further purification. To a solution of diethyl phosphite (0.275 ml, 2.13 mmol, 1.20 eq) in 12 ml THF was added LiHMDS (2.13 ml, 2.13 mmol, 1.20 eq, 1 M in THF) dropwise at -78°C. After 15 min, a solution of the residue (1.32 g, 1.77 mmol, 1.00 eq) in 2.9 ml THF was added and the resulting mixture was stirred at -78°C for 1h, then quenched by addition of 6.5 ml saturated NH_4_Cl and 63 ml diethyl ether. The reaction mixture was stirred 15 min at -78°C and was then warmed up to RT. 8.5 ml of water was added and the organic layer was separated.  The aqueous layer was extracted with 2x21 ml diethyl ether and the combined organic fractions were dried over Na_2_SO_4_, filtered and concentrated. The residue was purified by flash column chromatography on silica gel (DCM/MeOH 98:2 to 96:4 to 94:6) to yield a major stereoisomer (1.10 g, 1.24 mmol, 70 %, Rf = 0.46, DCM/MeOH 94:6) and a minor stereoisomer of **31** (128 mg, 0.15 mmol, 8 %, Rf = 0.38, DCM/MeOH 94:6) in a 8.6:1 ratio as colorless solids. ^1^H NMR major dia (400 MHz, CDCl_3_) δ = 7.29-7.32 (m, 10H, Ar-H), 7.19 (d, *J* = 10.0 Hz, 1H, NH), 5.75 (d, *J* = 8.4 Hz, 1H, NH), 5.07-5.12 (m, 2H), 4.97-5.02 (m, 1H), 4.65 (d, *J* = 11.6 Hz, 1H), 4.45-4.52 (m, 3H), 4.27-4.32 (m, 2H), 4.16-4.22 (m, 4H), 4.05-4.12 (m, 4H), 3.99 (t, *J* = 8.8 Hz, 1H), 3.90-3.91 (m, 2H), 3.80-3.83 (m, 2H), 3.69-3.73 (m, 1H), 3.57-3.60 (m, 1H), 2.22 (s, 3H, NHAc), 2.05, 2x2.03 (3xs, 3x3H, 3xOAc), 1.92 (s, 3H, NHAc), 1.26 (dt, *J* = 14.4 Hz, 7.2 Hz, 6H, 2xOCH_2_CH_3_). ^13^C NMR major dia (101 MHz, CDCl_3_) δ = 172.9, 170.5, 170.4, 169.6, 168.2 (5xs, 3xOC=OCH_3_+ 2xNHC=OCH_3_), 137.2, 136.1, 127.5, 127.4, 127.0, 126.9, 126.5 (7xs, 7xAr-C), 99.7, 74.2, 73.6, 72.7, 71.3, 71.2, 71.1, 70.8, 68.0, 67.0, 66.9, 66.4, 64.7, 62.0, 62.0, 61.0, 61.0, 60.9, 60.8, 52.6, 44.2, 44.1, 22.3, 21.7 (2xs, 2xNHC=OCH_3_), 19.7, 19.6, 19.6 (3xs, 3xOC=OCH_3_), 15.5, 15.5 (2xs, 2x OCH_2_CH_3_). ^31^P NMR major dia (122 MHz, CDCl_3_) δ = 24.6. ESI-HRMS major dia (+) *m/z* calculated 881.3468 (M+[H^+^]), found 881.3442 for C_41_H_58_N_2_O_17_P^+^. ^1^H NMR minor dia (400 MHz, CDCl_3_) δ = 7.26-7.32 (m, 10H, Ar-H), 7.12 (d, *J* = 10.0 Hz, 1H, NH), 6.01 (d, *J* = 9.2 Hz, 1H, NH), 4.98-5.09 (m, 3H), 4.71 (d, *J* = 11.6 Hz, 1H), 4.42-4.56 (m, 5H), 4.27-4.32 (m, 3H), 4.01-4.17 (m, 8H), 3.83-3.87 (m, 1H), 3.73-3.79 (m, 2H), 3.56-3.60 (m, 1H), 2.09, 2.05, 2.02, 2.00, 1.95 (5xs, 5x3H, 3xOAc + 2xNHAc), 1.23-1.27 (m, 6H, 2xOCH_2_CH_3_). ^13^C NMR minor dia (101 MHz, CDCl_3_) δ = 170.6, 170.5, 170.2, 169.6, 168.2 (5xs, 3xOC=OCH_3_+ 2xNHC=OCH_3_), 136.9, 136.7, 127.5, 127.5, 127.4, 127.0, 126.9, 126.8, 126.4 (9xs, 9xAr-C), 99.8, 74.9, 74.3, 72.7, 71.9, 71.1, 71.1, 70.9, 68.7, 67.1, 66.4, 65.8, 65.7, 62.0, 61.9, 61.4, 61.4, 60.9, 53.2, 45.7, 45.6, 43.6, 22.5, 22.1 (2xs, 2xNHC=OCH_3_), 19.8, 19.7, 19.6 (3xs, 3xOC=OCH_3_), 15.5, 15.4 (2xs, 2x OCH_2_CH_3_). ^31^P NMR minor dia (122 MHz, CDCl_3_) δ = 21.5. ESI-HRMS minor dia (+) *m/z* calculated 903.3287 (M+[Na^+^]), found 903.3259 for C_41_H_57_N_2_NaO_17_P^+^.

**(2R,3S,4R,5R,6S)-5-acetamido-6-(((2R,3S,4R,5R,6S)-5-acetamido-4-(benzyloxy)-2-((benzyloxy)methyl)-6-((diethoxyphosphoryl)methyl)tetrahydro-2H-pyran-3-yl)oxy)-2-(acetoxymethyl)tetrahydro-2H-pyran-3,4-diyl diacetate (32)**

To a solution of **31** (major dia) (200 mg, 0.23 mmol, 1.00 eq), 4 Å crushed molecular sieves in 5.5 ml dry DCM was added pyridine (0.18 ml, 2.27 mmol, 10.00 eq), DMAP (55.5 mg, 0.45 mmol, 2.00 eq) and methyl oxalyl chloride (0.10 ml, 1.14 mmol, 5.00 eq) respectively. The reaction mixture was stirred at RT under an Ar atmosphere overnight, filtered on celite, acidified to pH 1 with 1M HCl and distilled water was added. The phases were separated and the aqueous phase was extracted with DCM. The combined organic phases were washed with brine, dried over Na_2_SO_4_, filtered and concentrated to dryness. The residue was dissolved 2 ml toluene and Bu_3_SnH (0.27 ml, 1.00 mmol, 5.00 eq) and AIBN (4.9 mg, 0.03 mmol, 0.15 eq) were added. The reaction mixture was heated to reflux and stirred for 3h and purified by flash column chromatography on silica gel (DCM/MeOH 97:3 to 96:4, Rf = 0.17, DCM/MeOH 96:4) to yield **32** (127 mg, 0.15 mmol, 65 %) as a colorless solid. ^1^H NMR (400 MHz, CDCl_3_) δ = 7.26-7.32 (m, 10H, Ar-H), 7.06 (d, *J* = 10.4 Hz, 1H, NH), 6.04 (d, *J* = 9.2 Hz, 1H, NH), 5.03-5.10 (m, 3H), 4.69 (d, *J* = 11.6 Hz, 1H), 4.47-4.63 (m, 3H), 4.36-4.41 (m, 1H), 4.21-4.30 (m, 3H), 4.12 (t, *J* = 9.2 Hz, 1H), 3.99-4.07 (m, 4H), 3.86-3.89 (m, 2H), 3.78-3.84 (m, 2H), 3.72 (s, 1H), 3.57-3.61 (m, 1H), 2.05, 2.01, 2.01, 2.00, 1.96 (5xs, 5x3H, 3xOAc + 2xNHAc), 1.90-1.94 (m, 2H, C1-CH_2_-P), 1.23 (dt, *J* = 13.2 Hz, 7.2 Hz, 6H, 2xOCH_2_CH_3_). ^13^C NMR (101 MHz, CDCl_3_) δ = 170.6, 170.5, 170.3, 169.6, 168.2 (5xs, 3xOC=OCH_3_+ 2xNHC=OCH_3_), 137.1, 136.6, 127.4, 127.4, 126.9, 126.8, 126.8, 126.4 (8xs, 8xAr-C), 99.7, 74.4, 74.4, 72.7, 71.2, 71.1, 71.0, 70.9, 67.2, 66.9, 63.1, 63.1, 60.9, 60.8, 60.7, 60.3, 60.2, 52.8, 46.3, 46.1, 28.6, 27.2, 26.9, 25.8, 22.5, 21.9 (2xs, 2xNHC=OCH_3_), 19.8, 19.7, 19.6 (3xs, 3xOC=OCH_3_), 15.4 15.4 (2xd, *J* = 2x5.1 Hz, 2x OCH_2_CH_3_). ^31^P NMR (122 MHz, CDCl_3_) δ = 29.6. ESI-HRMS (+) *m/z* calculated 865.3518 (M+[H^+^]), found 865.3513 for C_41_H_58_N_2_O_16_P^+^.

**(((2S,3R,4R,5S,6R)-3-acetamido-5-(((2S,3R,4R,5S,6R)-3-acetamido-4,5-diacetoxy-6-(acetoxymethyl)tetrahydro-2H-pyran-2-yl)oxy)-4-hydroxy-6-(hydroxymethyl)tetrahydro-2H-pyran-2-yl)methyl)phosphonic acid (33)**

**32** (102 mg, 0.12 mmol, 1.00 eq) was dissolved in 5 ml MeOH and Pd(OH)_2_/C (33.10 mg, 0.24 mmol, 2.00 eq) was added. The reaction mixture was put with a needle under vacuum and hydrogen atmosphere 3 times and was then stirred under hydrogen atmosphere for 6h. 2 times 1 more eq of Pd(OH)_2_/C were added to have a total conversion. Pd was filtered off on celite and the solvent was removed under reduced pressure. The dry residue was dissolved in 2 ml dry DCM and TMSBr (0.31 ml, 2.36 mmol, 20.00 eq) was added dropwise at RT under an Ar atmosphere. The reaction mixture was stirred for 1.5h and then 20 more eq of TMSBr were added. As the reaction didn't go to completion, 20 more eq of TMSBr were added at 18h and the day after at 8h. The reaction mixture was concentrated *in vacuo*, dissolved in 5 ml acetone and 0.30 ml H_2_O was added. The reaction mixture was stirred at RT for 30 min and then concentrated to dryness to yield **33** (74 mg, 0.12 mmol, quantitative yield over 2 steps) as an orange solid which was used in the next step without further purification.

**(((2S,3R,4R,5S,6R)-3-acetamido-5-(((2S,3R,4R,5S,6R)-3-acetamido-4,5-dihydroxy-6-(hydroxymethyl)tetrahydro-2H-pyran-2-yl)oxy)-4-hydroxy-6-(hydroxymethyl)tetrahydro-2H-pyran-2-yl)methyl)phosphonic ((2E,6E)-3,7,11-trimethyldodeca-2,6,10-trien-1-yl phosphoric) anhydride (34b)**

**16b** (91.5 mg, 0.27 mmol, 3.00 eq) and CDI (221 mg, 1.36 mmol, 15.00 eq) were dissolved in 2.5 ml DMF and stirred for 3 h at RT under an Ar atmosphere. 0.10 ml dry MeOH was added and the reaction mixture was stirred for 1 h to quench the unreacted CDI.
MeOH was removed *in vacuo* and **33** (57 mg, 0.09 mmol, 1.00 eq) in 1 ml DMF was added to the reaction mixture which was then stirred for 6 days at RT under an Ar atmosphere. The reaction mixture was concentrated *in vacuo*. The residue was dissolved in 20 ml MeOH and ammonium hydroxyde (8.67 ml, 54.40 mmol, 600 eq) was added. The reaction mixture was stirred at RT overnight, then concentrated at the rotavapor and freeze-dried. The residue was purified by flash column chromatography on basified silica gel with 25 % ammoniac solution in water (EtOAc/iPrOH/H_2_O 4:2:1, Rf = 0.09) to yield **34b** (19.6 mg, 0.02 mmol, 26 % over 2 steps) as a colorless lyophilisat. ^1^H NMR (400 MHz, MeOD) δ = 5.43 (t, *J* = 6.4 Hz, 1H, H-2’), 5.08-5.15 (m, 2H, H-6’, H-10’), 4.51 (t, *J* = 6.4 Hz, 3H, H-1’,H-1a), 4.44 (d, *J* = 8.4 Hz, 1H, H-5a), 4.01 (dd, *J* = 7.6 Hz, 4.4 Hz, 1H, H-2a), 3.88-3.91 (m, 2H, H-6b1, H-6b2), 3.79-3.84 (m, 2H, H-3a, H-5b), 3.64-3.72 (m, 3H, H-6a1, H-6a2, H-4b), 3.44-3.51 (m, 2H, H-4a, H-3b), 3.33-3.35 (m, 2H, H-1b, H-2b), 1.94-2.15 (m, 16H, H-4’, H-5’, H-8’, H-9’, H-7, 2xNHAc), 1.70 (s, 3H, H-12’), 1.67 (s, 3H, H-13’), 1.61 (s, 6H, H-14’, H-15’). ^13^C NMR (101 MHz, MeOD) δ = 172.9, 172.2 (2xs, 2xNHC=OCH_3_), 139.3 (s, C-3’), 134.8 (s, C-7’), 130.7 (s, C-11’), 124.0 (s, C-10’), 123.8 (s, C-6’), 121.2 (s, C-2’), 101.3 (s, C-5a), 78.9 (s, C-4a), 76.7 (s, C-1b), 74.2 (s, C-3b), 73.9 (s, C-5b), 70.6 (s, C-2b), 69.0 (s, C-3a), 67.3 (s, C-1a), 62.4 (d, *J* = 5.5 Hz, C-1’), 61.2 (s, C-6b), 60.0 (s, C-6a), 56.0 (s, C-4b), 52.6 (s, C-2a), 39.4 (d, *J* = 16.7 Hz, C-7), 2x26.4, 2x26.1 (4xs, C-4’, C-5’, C-8’, C-9’), 24.5 (s, C-13’), 22.0, 21.4 (2xs, 2xNHC=OCH_3_), 16.4, 15.2 (2xs, C-14’, C-15’), 14.7 (s, C-12’). ^31^P NMR (122 MHz, MeOD) δ = 13.3 (d, *J* = 25.4 Hz, P1), -10.3 (d, *J* = 23.9 Hz, P2). ESI-HRMS (-) *m/z* calculated 785.3032 (M-[H^+^]), found 785.3037 for C_32_H_55_N_2_O_16_P_2_^-^.

**(((2S,3R,4R,5S,6R)-3-acetamido-5-(((2S,3R,4R,5S,6R)-3-acetamido-4,5-dihydroxy-6-(hydroxymethyl)tetrahydro-2H-pyran-2-yl)oxy)-4-hydroxy-6-(hydroxymethyl)tetrahydro-2H-pyran-2-yl)methyl)phosphonic ((S,6Z,10E,14E)-3,7,11,15,19-pentamethylicosa-6,10,14,18-tetraen-1-yl phosphoric) anhydride (34d)**

Following exactly the same procedure as for **34b** and starting from **16d** (72 mg, 0.15 mmol, 2.00 eq) and using CDI (123 mg, 0.76 mmol, 10.00 eq), **33** (47.5 mg, 0.08 mmol, 1.00 eq) and ammonium hydroxyde (7.22 ml, 45.30 mmol, 600 eq), we obtained **34d** (32 mg, 0.03 mmol, 44 % over 2 steps) as a colorless lyophilisat after freeze-drying (EtOAc/iPrOH/H_2_O 4:2:1, Rf = 0.10). ^1^H NMR (400 MHz, MeOD) δ = 5.08-5.14 (m, 4H, H-6’, H-10’, H-14’, H-18’), 4.50-4.56 (m, 1H, H-1a), 4.46 (d, *J* = 8.4 Hz, 1H, H-5a), 3.96-4.03 (m, 3H, H-1’, H-2a), 3.91 (dd, *J* = 11.2 Hz, 0.8 Hz, 1H, H-6b1), 3.84-3.85 (m, 2H, H-6b2, H-5b), 3.79 (t, *J* = 8.8 Hz, 1H, H-3a), 3.68-3.74 (m, 2H, H-6a1, H-5b), 3.65 (t, *J* = 6.0 Hz, 1H, H-6a2), 3.49 (dd, *J* = 11.0 Hz, 8.4 Hz, 1H, H-3b), 3.41 (t, *J* = 6.0 Hz, 1H, H-5a), 3.33-3.35 (m, 2H, H-1b, H-2b), 1.99-2.08 (m, 15H, H-7a, H-5’, H-8’, H-9’, H-12’, H-13’, H-16’, H-17’), 1.92 (s, 6H, 2xNHAc), 1.82-1.87 (m, 1H, H-7b), 1.71-1.74 (m, 1H, H-2’a), 1.67 (s, 6H, H-21’, H-22’), 1.60 (2xs, 10H, H-3’, H-23’, H-24’, H-25’), 1.41-1.46 (m, 1H, H-2’b), 1.35-1.39 (m, 1H, H-4’a), 1.15-1.19 (m, 1H, H-4’b), 0.93 (d, *J* = 6.4 Hz, 3H, H-20’). ^13^C NMR (101 MHz, MeOD) δ = 178.8, 174.1 (2xs, 2xNHC=OCH_3_), 136.0 (s, C-7’), 135.9 (s, C-11’), 135.9 (s, C-15’), 132.0 (s, C-19’), 126.6 (s, C-18’), 125.5 (s, C-14’), 125.4 (s, C-10’), 125.4 (s, C-6’), 102.8 (s, C-5a), 80.9 (s, C-4a), 78,1 (s, C-1b), 75.6 (s, C-3b), 75.0 (s, C-5b), 72.0 (s, C-2b), 70.2 (s, C-3a), 69.1 (s, C-1a), 65.4 (d, *J* = 6.3 Hz, C-1’), 62.6 (s, C-6b), 61.7 (s, C-6a), 57.4 (s, C-4b), 54.0 (s, C-2a), 40.9 (s, C-4’), 40.8 (s, C-8’), 38.9 (d, *J* = 12.4 Hz, C-7), 32.9 (s, C-12’), 30.6 (s, C-3’), 27.8 (s, C-16’), 27.6 (s, C-5’), 27.6 (s, C-9’), 26.4 (s, C-13’), 25.9 (s, C-25’), 23.7 (s, C-24’), 23.3 (s, NHC=OCH_3_), 23.1 (s, C-17’), 22.8 (s, NHC=OCH_3_), 19.8 (s, C-20’), 17.8 (s, C-23’), 16.1 (s, C-22’), 16.1 (s, C-21’). ^31^P NMR (122 MHz, MeOD) δ = 14.0 (d, *J* = 24.1 Hz, P1), -9.3 (d, *J* = 23.9 Hz, P2). ESI-HRMS (-) *m/z* calculated 923.4441 (M-[H^+^]), found 923.4466 for C_42_H_73_N_2_O_16_P_2_^-^.
